# Effectiveness of eHealth Interventions for Adolescents and Young Adults With Congenital Heart Disease: Systematic Review

**DOI:** 10.2196/91424

**Published:** 2026-07-08

**Authors:** Brith Andresen, Mari Oma Ohnstad, Marie Hamilton Larsen, Simen A Steindal, Henrik Brun, Katrine Onshuus Eriksen, Kristin Hofsø, Unni Jenssen, Marte-Marie Wallander Karlsen, Monica Evelyn Kvande, Kari L Mariussen, Philip Moons, Clarisse Sifa Nsengi, Kristin J Skaarud, Karoline Skedsmo, Kari Sørensen, Benedicte S Strøm, Ina Thon Aamodt

**Affiliations:** 1 Department of Master and Postgraduate Studies Lovisenberg Diaconal University College Oslo Norway; 2 Department of Cardiothoracic Surgery Oslo University Hospital Oslo Norway; 3 Institute of Nursing, Faculty of Health VID Specialized University Oslo Norway; 4 Institute of Clinical Medicine University of Oslo Oslo Norway; 5 Department of Pediatric Cardiology Oslo University Hospital Oslo Norway; 6 The Intervention Centre Oslo University Hospital Oslo, Oslo Norway; 7 Clinic of Cardiology St Olav's University Hospital Trondheim Norway; 8 Department of Postoperative and Critical Care Nursing, Division of Emergencies and Critical Care Oslo University Hospital Oslo Norway; 9 Lovisenberg Diaconal University College Oslo Norway; 10 KU Leuven Department of Public Health and Primary Care University of Leuven Leuven Belgium; 11 University of Gothenburg Centre for Person-Centred Care (GPCC), Sahlgrenska Academy University of Gothenburg Gothenburg Sweden; 12 Department of Paediatrics and Child Health University of Cape Town Cape Town South Africa; 13 Department of Haematology Oslo University Hospital Oslo Norway; 14 Department of Otorhinolaryngology, Head and Neck Oslo University Hospital Oslo Norway; 15 Department of Nursing and Health Promotion, Faculty of Health Sciences Oslo Metropolitan University Oslo Norway

**Keywords:** adolescents and young adults, congenital heart disease, eHealth, health care professionals, peer support, self-management, systematic review

## Abstract

**Background:**

Adolescents and young adults (AYAs) with complex congenital heart disease (CHD) may benefit from eHealth interventions, particularly when early signs of deterioration or self-management challenges arise. eHealth can enhance self-management skills and communication with health care professionals (HCPs). However, systematic reviews examining eHealth interventions for this population remain limited and heterogeneous, indicating a need for a systematic review of the literature to guide the development, implementation, and evaluation of such interventions.

**Objective:**

In this systematic review, we aimed to synthesize the content, theoretical foundations, outcomes, and effectiveness of eHealth interventions targeting AYAs with complex CHD that involve interaction with HCPs.

**Methods:**

We conducted this systematic review in accordance with the Cochrane Handbook for Systematic Reviews of Interventions. An a priori protocol was registered in PROSPERO (CRD42023400211). The review was reported according to the 2020 PRISMA (Preferred Reporting Items for Systematic Reviews and Meta-Analyses), the PRISMA-S (PRISMA Literature Search Extension), and the SWiM (Synthesis Without Meta-Analysis) checklists. The final comprehensive bibliographic search was conducted on March 16, 2026, across MEDLINE ALL (Ovid), Embase (Ovid), APA PsycInfo (Ovid), ERIC (EBSCOhost), CINAHL (EBSCOhost), and the Web of Science Core Collection (Clarivate). Studies were eligible if they included AYAs aged 10-29 years diagnosed with CHD who received eHealth interventions delivered by HCPs regardless of care setting; measured effects of eHealth interventions using experimental or quasiexperimental designs; and were published in English or a Scandinavian language. The researchers assessed eligibility in pairs, risk of bias using the Revised Cochrane risk-of-bias tool for randomized trials (RoB 2) for randomized studies and the Risk of Bias in Non-Randomized Studies of Exposures (ROBINS-E) for nonrandomized studies, and extracted data. Overall risk of bias was high. Due to heterogeneity among the included studies, a narrative synthesis was conducted.

**Results:**

We included 8 studies, with sample sizes ranging from 20 to 158, totaling 551 participants. Five studies reported improvements in the eHealth intervention groups, evaluating educational interventions to improve disease-specific knowledge, self-management, physical activity, psychosocial impact, and transition readiness in AYAs with CHD. However, the content, delivery mode and format, and target outcomes varied across studies. Seven studies lacked a theoretical foundation for the intervention.

**Conclusions:**

The evidence remains inconsistent and limited by heterogeneity, absence of theoretical foundations, risk of bias, and high dropout rates; further refinement is thus needed. Future research may benefit from theory-driven, user-centered, co-designed approaches with AYAs with CHD from the outset to enhance relevance, engagement, and long-term sustainability. Standardized outcome measures and long-term evaluations could clarify the long-term impact and implementation potential of eHealth interventions. This study emphasizes factors like tailoring content to medical complexity, integrating interactive interprofessional teams, and incorporating structured peer support in planning and developing eHealth intervention research in the future.

## Introduction

### Overview

Congenital heart disease (CHD) occurs when the heart or blood vessels close to the heart do not develop normally before birth. Mitchell et al [1] define CHD as “a gross structural abnormality of the heart or intrathoracic great vessels that is actually or potentially of functional significance.” The global prevalence of CHD is reported to be 0.8% to 1% [2]. The severity of CHD ranges from simple to highly complex conditions [3], categorized as simple, moderate, or complex [4]. Approximately 30% of newborns with CHD have multiple structural defects in their hearts, classified as patients with complex CHD [5]. CHD conditions are highly individual, and the anatomical morphology can vary significantly within the same diagnostic category [6]. Surgical and interventional corrections often require supplementary tissue in different formats, such as patches, conduits, and valves [7]. Structural complications, degeneration, calcification, or insufficient growth may lead to multiple reinterventions due to the necessary replacement of implants during a patient’s lifespan.

The transition from childhood to adolescence and young adulthood, defined as spanning ages 10 to 29 years [8], represents a particularly vulnerable developmental phase. During this period, individuals experience identity formation and emotional development, develop greater independence, and assume increasing responsibility for their personal health; they also take on new social roles in education or employment settings [9,10]. In addition to attending regular examinations and follow-up visits in health care settings [11], they assume greater responsibility for adhering to medical prescriptions, diet, and exercise routines—behaviors essential to improving and maintaining good health [12]. As adolescents’ understanding of their illness evolves, they often become more actively involved in managing their health, facilitating a successful transition to adult care and chronic disease management [13]. Early recognition and assessment of symptoms in chronic diseases not only help preserve health and reduce mortality but also frequently require consistent health care services [10]. For adolescents and young adults (AYAs) with complex CHD, successful transition to adult care depends on disease knowledge, self-management skills, and timely recognition of symptoms. Furthermore, AYAs with CHD may experience parental overprotection and restrictions on physical activity, emphasizing the importance of fostering their confidence and autonomy during the transition from pediatric to adult health care [14]*.* As a population requiring lifelong clinical surveillance, adolescents growing up in the digital era may benefit from eHealth interventions, particularly when early signs of health deterioration or challenges in self-management arise [15]. Consequently, such interventions may help support adolescents’ growing independence.

### eHealth in the Context of CHD

The World Health Organization (WHO) defines eHealth as “the use of information and communication technologies for health,” including digital platforms, mobile apps, wearable devices, and telecommunication services [16]. We define eHealth interventions as digitally delivered programs designed to support health management, where at least 1 component involves active interaction between the AYAs and a health care professional (HCP). AYAs have reported that they prefer to share information and discuss their concerns with HCPs, in line with Dwyer-Matzky et al [17], who found that 50% of AYAs with chronic diseases wanted to establish close relationships with medical teams. This interaction can be synchronous (eg, videoconferencing) or asynchronous (eg, personalized feedback messages and in-app chats) but must include 2-way communication. Such interventions may address multiple needs of AYAs with complex CHD, including disease education, physical activity promotion, transition readiness, and coping strategies. They also hold the potential to reduce geographical barriers and enhance continuity of care [4,18], since long travel distances to specialized centers and interruptions in follow-up care can contribute to deterioration in health status [19].

A systematic review and meta-analysis evaluated the effectiveness of eHealth interventions in patients with chronic disease (eg, heart failure). The results suggest improvement in all-cause mortality, heart failure–related readmission, heart failure knowledge, quality of life, and self-care [20]. A previous systematic review [21] explored the effectiveness of mobile apps to support adolescents’ management of their physical chronic condition, finding that evidence-based apps with active input between users and HCPs were lacking. Kauw et al [22] found that home monitoring of oxygen saturation and regular weight control were beneficial among children and adolescents with CHD after corrective cardiothoracic surgery. In addition, videoconferences had a positive effect on anxiety levels and health care use. However, the review by Kauw et al [22] had several limitations, including an unclear search strategy, reliance on a single database, and a lack of quality assessment of the included studies. Furthermore, the results were heterogeneous across outcome measures in various settings, making it difficult to draw conclusions about the effects.

Despite initial database searches, we were unable to identify any systematic reviews specifically examining eHealth interventions for AYAs with CHD conditions that involve interaction with HCPs. The effectiveness of these interventions often depends on the availability and consistency of health care services, which remain variable across different populations and settings. With the expansion of technology-based care, quantitative evidence regarding determinants and health outcomes on self-management skills in AYAs with CHD in communication with HCPs is sparse and fragmented; a comprehensive synthesis is therefore needed to further guide intervention development, implementation, and evaluation.

The objective of this systematic review was to synthesize the content, theoretical foundation, outcomes, and effectiveness of eHealth interventions targeting AYAs with CHD that involve interaction with HCPs.

## Methods

### Study Design and Registration

This systematic review was conducted in accordance with the Cochrane Handbook for Systematic Reviews of Interventions. The reporting of this review followed the 2020 PRISMA (Preferred Reporting Items for Systematic Reviews and Meta-Analyses; Multimedia Appendix 1) checklist [23], the PRISMA-S (PRISMA Literature Search Extension; Multimedia Appendix 2) checklist and supporting materials from the PRISMA website (PRISMA Group, 2020; accessed April 23, 2026) to guide the presentation of methods and results [24], and the SWiM (Synthesis Without Meta-Analysis; Multimedia Appendix 3) checklist for reporting items [25]. An a priori protocol was registered in PROSPERO (CRD42023400211) [26]. The research team consisted of 18 members, all with professional backgrounds in nursing, medicine, and research, as well as one experienced academic librarian.

### Eligibility Criteria

Eligibility criteria were structured according to the Population, Intervention, Comparison, and Outcome (PICO) framework [27], supplemented by specifications regarding study design, language, and publication period (Table 1).

**Table 1:** Eligibility criteria based on the PICOS^a^ framework.

Criterion	Inclusion	Exclusion
Population (P)	AYAs^b^—defined as being aged 10-29 years and diagnosed with CHD^c^	AYAs with other heart conditions or chronic diseases and adults (aged ≤30 years), family members or parents of adolescents with CHD, and HCPs^d^ caring for adolescents with CHD
Interventions (I)	eHealth interventions delivered by HCPs regardless of care setting. eHealth interventions are defined as digitally delivered programs designed to support health management, where at least 1 component involves active interaction between the AYAs and an HCP. This interaction can be synchronous (eg, videoconferencing) or asynchronous (eg, personalized feedback messages and in-app chat) but must include the possibility for 2-way communication	N/A^e^
Comparisons (C)	Any kind of comparator: for example, treatment control groups, waitlist control groups, attention control groups (participants receive some other attention), or standard care control groups; pre-post comparisons	No comparator
Outcomes (O)	All parameters measuring the effect of eHealth interventions	Outcomes not measuring the effect of eHealth interventions
Study design (S)	Experimental or quasiexperimental designs (eg, pre-post studies with a control group)	All other research designs or type of publication types
Language	English, Norwegian, Swedish, or Danish	All other languages
Period	After January 1, 2010	After March 16, 2026

^a^PICOS: Population, Interventions, Comparisons, Outcomes, and Study Design.

^b^AYA: adolescent and young adult.

^c^CHD: congenital heart disease.

^d^HCP: health care provider.

^e^N/A: not applicable.

### Information Sources

A comprehensive bibliographic search was conducted on April 27, 2023, across the following databases: MEDLINE ALL (Ovid), Embase (Ovid), APA PsycInfo (Ovid), ERIC (EBSCOhost), CINAHL (EBSCOhost), and the Web of Science Core Collection (Clarivate). An updated search across all databases was conducted on September 13, 2024, and March 16, 2026. The reference lists of included articles were hand-searched to identify potentially eligible studies not captured in the initial database search.

### Search Strategy

The search strategy was initially developed in MEDLINE ALL (Ovid) by an experienced librarian in collaboration with the first and last author. We conducted a comprehensive search combining controlled vocabulary (Medical Subject Headings [MeSH] terms) and free‑text keywords related to CHD and digital health interventions, including telemedicine, mobile health, remote monitoring, and related technologies. The search strategy was independently developed for this study without using pre-established search filters or existing search strings. The search was limited to studies published from 2010 onward in English, Norwegian, Danish, or Swedish, and excluded publication types such as editorials, letters, conference abstracts, and comments.

To reduce the risk of missing relevant studies, no attempt was made to limit the results to specific age groups. However, the databases’ built-in limits were applied for language and publication period, as were filters to exclude nonresearch publications such as comments, letters, editorials, and conference abstracts. No additional restrictions were applied regarding study design. The initial strategy was piloted by the first and last authors before being adopted for the remaining databases. It was subsequently peer-reviewed by a second librarian based on the Peer Review of Electronic Search Strategies checklist [28]. Full search strategies for all databases are provided in Multimedia Appendix 4.

### Selection Process

All identified records were imported to EndNote (Clarivate) for duplicate removal. The remaining references were then randomly divided into 7 groups and uploaded to Rayyan, a web-based tool for systematic reviews [29], using blind screening mode. Each group was assigned to a pair of researchers. The study selection was carried out in 2 phases.

Step 1: titles and abstracts were independently screened by pairs of researchers using the predefined eligibility criteria. After this initial screening, blind mode was deactivated to display the results of the screening, allowing the pairs of researchers to discuss and reach an agreement when there were conflicts within the pairs.

Step 2: articles included in step 1 were retrieved in full text, and full-text articles were independently assessed for eligibility by the same researcher pairs. Notably, the full-text articles assigned in step 2 were different from those screened by the same pairs in step 1 to reduce bias. In both steps, any disagreements regarding inclusion were resolved through discussion with the first author, who conducted an independent assessment. Final decisions were reached by consensus.

### Data Extraction

A standardized data extraction form was developed in Microsoft Excel to systematically collect the following information from each included study: first author’s last name, year of publication, country of origin, study design, sample size, age of the participants, intervention, theoretical perspective, measurement instruments, and reported outcomes and effectiveness of the eHealth intervention in relation to the review aim.

The extraction form was piloted by the first and last authors, who worked independently on 8 included studies to ensure consistency and clarity. Subsequently, data extraction was performed, where one researcher extracted data and the other verified accuracy against the full-text articles. Any discrepancies were resolved through discussion between the first and last authors and in agreement with all authors.

### Assessment of Risk of Bias

The risk of bias of the randomized controlled trials (RCTs) was assessed using the Revised Cochrane risk-of-bias tool for randomized trials (RoB 2) [30]. This tool assesses five domains of bias: (1) bias arising from the randomization process, (2) deviations from the intended interventions, (3) missing outcome data, (4) measurement of the outcome, and (5) selection of the reported results. Each study was assigned an overall risk-of-bias judgment—low risk, some concerns, or high risk—based on these domains.

For nonrandomized studies, the Risk of Bias in Non-Randomized Studies of Exposures (ROBINS-E) tool was applied [31]. This tool assesses seven domains (1) confounding, (2) measurement of the exposure, (3) selection of participants, (4) postexposure interventions, (5) missing data, (6) measurement of the outcome, and (7) selection of the reported results. Overall risk was categorized as low risk (except for potential uncontrolled confounding), some concerns, high risk, or very high risk [31].

The risk-of-bias assessment was independently conducted by 2 pairs of researchers (MHL, SAS, MOO, and KH). Evaluations followed the tool’s respective algorithm and structured templates. For each study, domain-specific judgments were combined to produce an overall risk-of-bias rating, following RoB 2 and ROBINS-E guidance [30,31].

### Data Synthesis

All extracted data are presented in a literature matrix in which each study is described by its characteristics (author, country of origin, study design, sample size, age, intervention content and delivery, theoretical perspective or theory, and outcomes), in a summary table (patient-reported outcomes), and in the main text in the Results section. Due to a small number of included studies and substantial clinical and methodological heterogeneity, data were insufficiently comparable for statistical pooling, and a meta-analysis was not performed. Therefore, we conducted a narrative synthesis of the included studies by synthesizing across-study patterns through text summary, counts, and range in accordance with the PRISMA-S 2020 checklist [24] provided in Multimedia Appendix 2. We highlighted consistent and divergent findings and interpreted results in light of each study’s risk of bias.

## Results

### Characteristics of Included Studies

**Figure 1:**
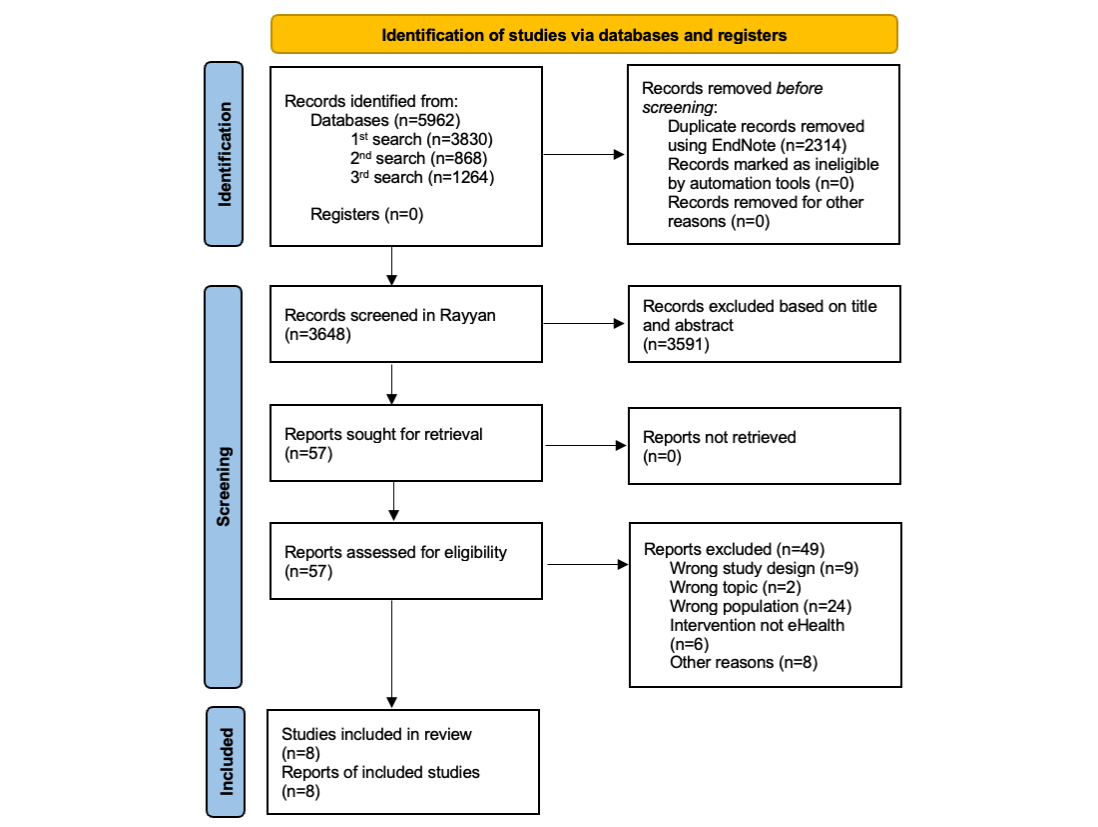
PRISMA (Preferred Reporting Items for Systematic Reviews and Meta-Analyses) flow diagram of study selection. This figure illustrates the process of identification, screening, eligibility, assessment, and inclusion of studies in the systematic review in accordance with PRISMA 2020 guidelines.

The initial and updated searches yielded a total of 5962 records. After removal of duplicates, a total of 3648 titles and abstracts were screened. Of these, 3591 were excluded, and 57 full-text articles were assessed for eligibility. The main reasons for exclusion at this stage were incorrect population, study design, or intervention type (ie, not eHealth-based). A total of 8 studies were included in the review (Figure 1).

Of the 8 included studies, 4 were conducted in the United States [32-35], 1 in Canada [36], 1 in Denmark [37], 1 in Taiwan [38], and 1 in South Korea [39]. The study designs included 5 RCTs [32,34,37-39], 1 feasibility RCT [34], and 2 pre-post intervention studies, 1 with a control group [33] and 1 single-group within-subject study [35] (Table S1 in Multimedia Appendix 5 [33,34,36-41]).A total of 551 AYAs participated across the 8 included studies, with sample sizes ranging from 20 to 158 participants, encompassing both male and female participants. All included studies involved participants aged 12-24 years, with most studies focusing on patients aged 12-19 years [32-37,39] and 1 study including participants aged 15-24 years [38]. Studies included AYAs diagnosed with simple [32,33,36,38], moderate [38,39], and complex CHD [32-37,39].

### Theoretical Foundations

One of the included 8 studies explicitly grounded their interventions or work within a formal theoretical framework called the WE BEAT Well-Being Education Program [35], developed with an overall objective to foster positive psychological well-being and resilient outcomes [35]. However, several studies were inspired by existing theories [39,42,43] or theory-informed tools [44]. Klausen et al [37] cited Bandura’s social cognitive theory [44] as a guiding framework in their work, while Han et al [36] referred to inspiration principles from educational psychology and used a theory-informed questionnaire as part of their data collection process [42]. Freedenberg et al [32] used a mindfulness-based stress reduction approach [45], while Jackson et al [34] applied the theory of planned behavior [43] as an inspiration. The study by Lin et al [38] was informed by a self-regulation theory [46,47], while Liddle et al [33] drew inspiration from educational approaches in prior research to structure their interventional sessions [48]. Hwang et al [39] based their eHealth management program on a self-efficacy theory [49].

### Content of the eHealth Intervention

All studies included education interventions aimed at enhancing disease comprehension and improving transition readiness. Han et al [36], Lin et al [38], and Hwang et al [39] addressed personal health self-management, such as understanding medications, endocarditis prevention, nutrition decisions, mood and feelings, health progress, and patient rights. Liddle et al [33] addressed medical knowledge, and Klausen et al [37], Jackson et al [34], Lin et al [38], and Hwang et al [39] addressed physical activity as part of their intervention. Freedenberg et al [40] targeted emotional and cognitive regulation related to stress management, while Cousino et al [35] addressed resilience and well-being through a psychoeducation and coping skills program (Multimedia Appendix 6).

### Digital Delivery Formats and Platforms

The delivery formats of the eHealth interventions varied across studies, from individual approaches (eg, personalized feedback, one-to-one sessions, and individualized tracking tools) [33,34,37,39] to group-based approaches (eg, peer support, group discussions, or shared learning components) [32,35,36,38,39]. One study used both individual- and group-based approaches [38].

The digital delivery modes used in the interventions included applications, web-based platforms, and videoconferencing, with all studies using a combination of these modes. Six of the interventions were delivered primarily via a mobile app [32,34,36-39], with web-based platforms [33,38,39] and videoconferencing [32,34,35,39] used as additional modes in some studies. Delivery methods combined both synchronous and asynchronous modes [32-35,37-39], except for 1 intervention that used exclusively asynchronous modes [36]. Session lengths ranged from 10 to 60 minutes, depending on the format and content. The duration of the interventions varied across studies: for example, the tele-education model of Liddle et al [38] offered a single prearranged session and Hwang et al [39] conducted a health management program over 6 months with each group participating for 4 weeks, while Freedenberg et al [32] and Jackson et al [34] offered repeated group sessions over several weeks. Cousino et al [35] provided repeated group sessions over 5 weeks.

### HCPs’ Involvement in the eHealth Intervention

Intervention sessions were led by a variety of HCPs, including nurses [36,37], a pediatric cardiologist [33], a behavioral interventionist [34], and an exercise physiologist [37]. In 4 studies, the qualifications or roles of the HCP leading the sessions were not clearly described [32,37-39]. Klausen et al [37] referred to the facilitator as a “health coach specializing in adolescents” or a “trained coach.” Cousino et al [35] used a licensed psychologist or a supervised limited-licensed psychology trainee to lead the group sessions. In the study by Han et al [36], the same registered nurse delivered the education intervention to both the intervention and the control groups to ensure consistency. In study by Hwang et al [39], one researcher conducted the group sessions and provided telephone follow-up; however, it was not specified who was responsible for the standard CHD medical follow-up. Overall, HCPs and research staff contributed in various ways to implementing the interventions and other study-related tasks.

### Family Involvement in the eHealth Intervention

A total of 2 studies offered optional parent involvement. Liddle et al [33] invited parents to attend educational sessions and then give a free-text option, whereas Lin et al [38] included parents by asking them to respond to a questionnaire.

### Outcome Measures and Measurement Instruments

The reported primary outcomes were disease knowledge, maximal oxygen uptake, change in transition readiness, anxiety, depression, illness-related stress, self-efficacy, health behavior, health-related quality of life, feasibility, acceptability, and preliminary effectiveness of a novel group-based telemedicine psychoeducation program aimed at supporting psychological well-being [32,33,37-39,41]. Secondary outcomes were feasibility, sedentary behavior, lifestyle change, and preliminary effectiveness of the WE BEAT group-based program to guide the design of a future adequately powered, multisite effectiveness–implementation trial [34,37,41]. A total of 3 studies [32,38,39] did not clearly distinguish between primary and secondary outcomes; all reported outcomes are thus presented as primary outcomes (Table 2). A variety of patient-reported outcomes [50] were used across the included studies (Table 2). Among these, the Pediatric Quality of Life Inventory Questionnaire for teens—both generic and disease-specific—was used to measure quality of life [39,51]. Psychological outcomes, such as anxiety and depression, were measured by the Hospital Anxiety and Depression Scale [52], while illness-related stress was examined using the Responses to Stress Questionnaire [53]. Cousino et al [35] used the Connor-Davidson Resilience Scale to measure resilience [54,55], the Benefit and Burden Scale for Children to measure the benefit and burden of chronic illness [56], and the National Institutes of Health Patient-Reported Outcomes Measurement Information System scales [57,58] to measure depressive symptoms, anxiety, peer relationships, and life satisfaction. Transition readiness was assessed in 2 studies using the TRANSITION-Q Questionnaire [59], while the Leuven Knowledge Questionnaire for Congenital Heart Disease [60] was used to measure disease knowledge. Hwang et al [39] used the Korean Self-Rated Abilities for Health Practices: Self-Efficacy Measure [61]; health behavior was assessed using an ActiGraph for objective measures of physical activity, sedentary behaviors, and nocturnal sleep [62]. A questionnaire developed by Biglino et al [48,63] was used to evaluate the effectiveness of 3D cardiac heart models as a communication tool. Han et al [36] developed the Just Track it! Questionnaire as an eHealth intervention to improve transition readiness and self-management, which included multiple-choice and open-ended items to assess self-management skills, app use, and perceived utility (Multimedia Appendix 7).

**Table 2:** Measured outcomes, instruments, and patient‑reported outcome measures (PROMs) in included studies.

Authors	Outcomes measured	Measurement tools/methods	PROMs
Freedenberg et al [32]	Anxiety, depression, illness-related stress, and coping	HADS^a^ and RSQ^b^	HADS and RSQ
Jackson et al [34]	MVPA^c^, sedentary behavior, and cardiorespiratory fitness	ActiGraph accelerometer (MVPA and sedentary behavior) and exercise stress test (VO_2_ peak^d^)	None
Klausen et al [37]	Cardiorespiratory fitness (VO_2_ peak), physical activity, and HRQoL^e^	Cycle ergometer exercise test (VO_2_ peak), ActiGraph accelerometer and validated questionnaire (physical activity), and PedsQL^f^ generic and disease-specific modules (HRQoL)	PedsQL (generic and disease-specific modules)
Lin et al [38]	Disease knowledge and physical activity	LKQCHD^g^ and IPAQ^h^ (Taiwan version)	LKQCHD and IPAQ
Liddle et al [33]	Medical knowledge of cardiac defects and surgeries	Preintervention and postintervention questionnaires (free-text responses) scored by blinded cardiologists using a structured medical knowledge classification system	None (observer-rated outcome based on patient responses)
Han et al [36]	Transition readiness, frequency of use, and perceived usefulness of the intervention	TRANSITION-Q Questionnaire (validated measure of self-management skills) and study-specific questionnaire assessing frequency of use and perceived usefulness of smartphone apps	TRANSITION-Q Questionnaire
Hwang et al [39]	Health self-efficacy, health behaviors (physical activity, sedentary behavior, and sleep), and HRQoL	K-SRAHP^i^ (health self-efficacy), PCQLI^j^ (HRQoL), ActiGraph accelerometer (physical activity and sleep), and self-reported sedentary behavior questionnaire	K-SRAHP and PCQLI
Cousino et al [35]	Resilience, benefit/burden of illness, depressive symptoms, anxiety, peer relationships, and life satisfaction	Connor-Davidson Resilience Scale, Benefit/Burden Scale for Children, and NIH PROMIS^k^ questionnaires (depressive symptoms, anxiety, peer relationships, and life satisfaction)	Connor-Davidson Resilience Scale, Benefit/Burden Scale for Children, and NIH PROMIS measures

^a^HADS: Hospital Anxiety and Depression Scale.

^b^RSQ: Responses to Stress Questionnaire.

^c^MVPA: moderate-to-vigorous physical activity.

^d^VO2 peak: peak oxygen uptake.

^e^HRQoL: health-related quality of life.

^f^PedsQL: Pediatric Quality of Life Inventory.

^g^LKQCHD: Leuven Knowledge Questionnaire for Congenital Heart Disease.

^h^IPAQ: International Physical Activity Questionnaire.

^i^K-SRAHP: Korean Self-Rated Abilities for Health Practices.

^j^PCQLI: Pediatric Cardiac Quality of Life Inventory.

^k^NIH PROMIS: National Institutes of Health Patient-Reported Outcomes Measurement Information System.

### Effects of the eHealth Interventions

A total of 4 studies [32-34,39] reported statistically significant effects in favor of the intervention groups. One of these studies found a significant improvement in disease-specific medical knowledge among AYAs with CHD following the educational intervention using patient-specific digital 3D heart models [33]. Another study found that both intervention groups (one participating in a mindfulness-based stress reduction group and the other in a video support group) showed significant reductions in illness-related distress. However, no significant difference between groups was observed in anxiety or depression scores [32]. Furthermore, one study found increased cardiorespiratory fitness among participants with Tetralogy of Fallot who were least physically active at baseline, following the video-based activity intervention [34]. One study found that an online health management program enhanced self-efficacy, health behavior, and psychosocial impact. There were no significant differences in sleep behavior between the 2 groups [39]. Klausen et al [37] demonstrated that the eHealth intervention did not improve medical knowledge, physical activity, or health-related quality of life. Moreover, 2 studies did not report significant between-group differences [36,38]. Lin et al [38] reported no significant effect on disease knowledge or physical activity following the eHealth intervention, whereas Han et al [36] reported no significant effect on transition readiness in AYAs using smartphone technology. Cousino et al [41] reported that the WE BEAT group-based program was both feasible and acceptable, demonstrating meaningful effects on increasing resiliency and decreasing depressive symptoms.

### Incentives and Reinforcement of the eHealth Intervention

A total of 4 studies incorporated behavioral reinforcement strategies [34,37-39]. Freedenberg et al [32] offered rewards in both intervention arms (in-person mindfulness-based stress reduction and video) and emphasized the application of coping techniques in real-life situations. Jackson et al [34] used motivational feedback and positive messaging to increase adherence to physical activity recommendations, while Klausen et al [37] and Hwang et al [39] provided individually tailored SMS text messages and phone coaching as part of a health coaching model. In the study by Liddle et al [33], participants were mailed a USB drive containing a 3D video of their heart model, including a digital file for printing the model, while Lin et al [38] used gaming, featuring 15 stages and awarding participants with game coins. The game used was Whac-A-Mole style: players used a padded mallet to hit targets that popped up at random, testing reaction speed and focus in a fast-paced, repetitive task.

### Dropout in the Included Studies

The attrition rates in the included studies varied from low to high. Hwang et al [39] presented the lowest dropout rate at 3.45%, while Freedenberg et al [32] reported an 18% dropout rate across all groups. Han et al [36] reported a 26% dropout rate at 6 months, while Liddle et al [33] observed a 28% dropout rate in a pre-post study. Lin et al [38] reported a monthly attrition rate of 13.7%-29.6% (eg, rate of activity) over 1 year, and Jackson et al [34] presented a 36% dropout rate. Overall, dropout rates in the included studies ranged from 18% to 36%, with variation in time and time intervals. Klausen et al [37] noted 72% and 79% completion rates in both the intervention and control groups (though only 57% met app compliance criteria). Cousino et al [41] reported an 87% completion rate in their pilot pre-post study.

### Quality Assessment

**Figure 2:**
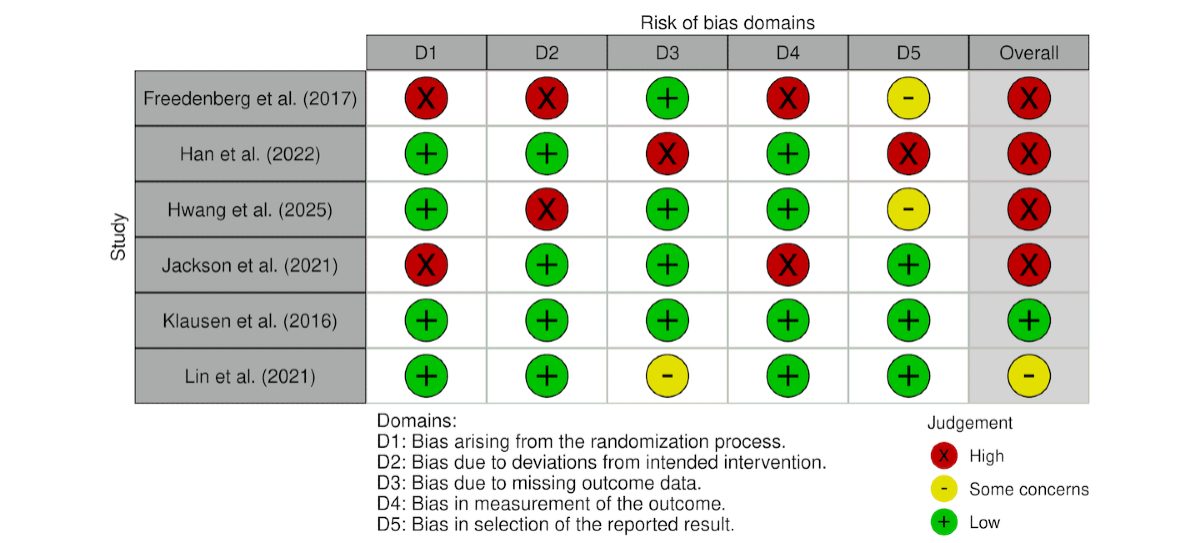
Traffic light plot of risk of bias domains across randomized controlled trial studies of Interventions [32,34,36,37,38,39] using Risk the Cochrane Risk of Bias tool (ROB-2).

**Figure 3:**
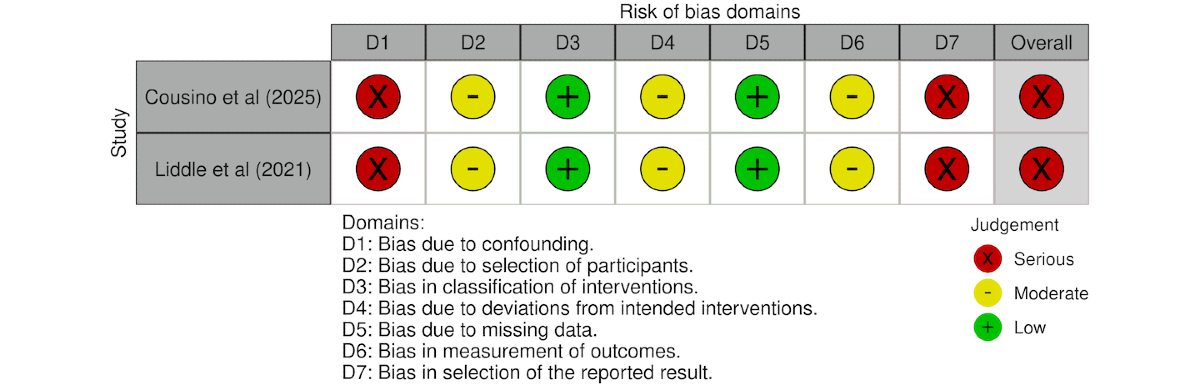
Traffic light plot of risk bias domains across pre-post studies [33,49] using Risk of Bias in Non-Randomized Studies of Interventions (ROBINS-I).

**Figure 4:**
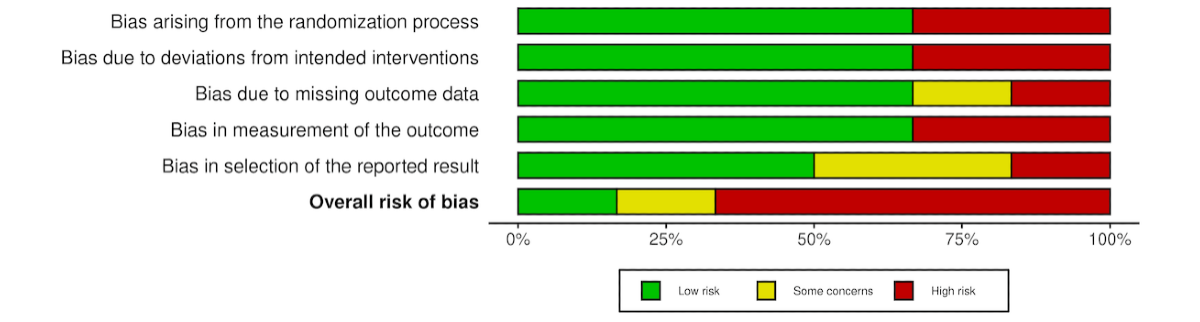
Summary of risk of bias assessments in all included studies.

The risk of bias varied across the included studies (Figures 2-4). A total of 6 studies were judged to have an overall high risk of bias [32-36,39], while 1 study had some concerns [38], and another was assessed as having a low risk of bias [37]. The domains that most frequently contributed to high risk were bias arising from the randomization process (D1) and bias in measurement of the outcome (D4), where several studies were judged as high risk. By contrast, bias due to missing outcome data (D3) was consistently low across most studies, except for the study by Han et al [36], which was rated high. Only the study by Klausen et al [37] received low-risk ratings across all domains. These findings indicate that the majority of the included studies had methodological limitations, particularly in relation to randomization and outcome measurement, which should be considered when interpreting the results of this review.

## Discussion

### Overview

The objective of this systematic review was to synthesize the content, theoretical foundations, outcomes, and effectiveness of eHealth interventions for AYAs with CHD that involved interaction with HCPs. Across the studies, eHealth interventions varied in content and delivery mode, showing promising findings; however, evidence for effectiveness and sustained improvement was inconsistent. Overall, a majority of the included studies reported improvements in disease-specific knowledge, self-management, physical activity, psychosocial impact, and transition readiness in AYAs with CHD when interventions combined educational and behavioral components. Few interventions were explicitly theory based, and substantial heterogeneity in the included studies limited direct comparisons.

### Effectiveness and Content of eHealth Interventions

The majority of studies in our systematic review [32-34,39] reported statistically significant effects on health education and self-management, suggesting that the interactive, multimodal design of these eHealth interventions correspond with the preferences of a population raised in a digital era—potentially stimulating a more engaging and motivational learning process than traditional education [15]. Such an approach may promote interventions tailored to meet each patient’s unique needs regarding age and disease complexity, while facilitating discussion about relevant challenges. It may also foster peer connection and support AYAs in taking greater responsibility for their own care [14,64].

While limitations in these studies included small sample sizes, clinical and methodological heterogeneity, moderate to high dropout rates, and an overall high risk of bias, our findings support the potential of eHealth interventions to help prevent adverse disease progression. AYAs with chronic and long-term conditions are at increased risk of developing traditional cardiovascular risk factors [65-67]; for instance, more than half of children and adolescents with surgically corrected heart defects have early signs of arteriosclerosis [68]. Multimodal communication may improve the acceptability of these interventions, engaging the AYAs while reducing attrition; however, the wide variation in these designs may partly explain the inconsistent findings regarding intervention effectiveness observed across studies.

The inclusion of multidimensional components in our systematic review—ranging from medication adherence and symptom monitoring to physical activity promotion and stress management—reflect an increasing recognition of the complex clinical and psychosocial needs of adolescents living with a chronic disease [15]. Adolescents with CHD are at increased risk of anxiety and depression, with reported high prevalence rates [69]. Previous research has described secondary control engagement coping skills (positive thinking, cognitive restructuring, acceptance, and distraction), associated with lower anxiety, depressive symptoms, somatic complaints, and withdrawal [70,71]. In our systematic review, positive messaging, tailored SMS text messages, and reward mechanisms were used to encourage healthy behaviors and coping skills, thus supporting participant engagement and adherence. In addition, the inclusion of gaming elements and digital visualization (eg, 3D heart models) illustrates innovative approaches to increase AYAs’ engagement. These strategies align with the developmental needs of AYAs, where interactive and rewarding tools may enhance both learning and sustained behavioral change [72]. However, only a subset of the studies included in our review used such strategies systematically, suggesting that behavioral reinforcement remains underused.

### Role of HCPs, Families, and Peers

The eHealth interventions in our review demonstrating improved effectiveness involved HCPs’ participation, although interprofessional collaboration and interaction among HCPs were generally lacking. In contrast to our review, a scoping review reported that HCPs using eHealth in home-based palliative care experienced feelings of improved collaboration, partnership, and peer support [73]. Similarly, Dwyer-Matzky et al [17] found that a majority of AYAs with chronic diseases wished to establish close connections with their medical teams—a factor associated with improved treatment adherence. Parental participation in virtual group interventions has also been shown to provide social support and improved communication with participants [33]. However, given the risk of noncompliance among AYAs, intervention designs may also benefit from allowing participants to identify accountability partners other than their caregivers, which may support autonomy and reinforce engagement with the intervention [15].

The integration of interprofessional teams through systematic interaction can improve implementation in clinical practice; this was highlighted in a scientific statement from the American Heart Association [4], where authors called for treatment strategies to prevent and treat heart failure in the pediatric population from a life perspective. To interact and communicate with this growing population, there is a need to accept eHealth as a potential supplement to outpatient follow-up services [16]. Guidelines for AYAs with CHD recommend the consideration of eHealth solutions in clinical practice [4,8]; however, the implementation of such interventions remains limited [18]. There thus appears to be a need to educate HCPs within interprofessional teams on the use of eHealth to facilitate its implementation into clinical practice [74].

### Theoretical Foundations

Notably, almost none of the included studies in our review explicitly grounded their intervention in a theoretical framework. When researching complex interventions, such as eHealth, using a theoretical framework can aid in understanding the underlying mechanisms driving their effectiveness [75]. Furthermore, applying theory during intervention development is essential for explaining observed effects and is associated with positive outcomes and larger effect sizes for increased clarity [75]. Grounding interventions in theory can also increase their relevance, support participant autonomy, and enhance long-term engagement [64,76]. The lack of theoretical frameworks may have influenced the definition of primary and secondary outcomes in our review, contributing to the heterogeneity observed across study design, intervention types, and measurement tools [77]. Moreover, none of the included studies in our review presented a definition of eHealth; this may have contributed to a heterogeneous understanding of the concept, consequently influencing the heterogeneity in outcomes. Future studies should define eHealth within their interventions to enhance consistency across study findings [22].

### Methodological Limitations of Included Studies

Although eHealth interventions should be designed to closely align with the needs of the population, none of the studies in our review reported a structured co-design process. Similar findings were reported by Li et al [15]. Previous research [76,77] has demonstrated that involving target users from the outset increases relevance, supports autonomy, and enhances long-term engagement. By actively engaging AYAs with CHD in defining priorities, carefully selecting intervention components (such as peer support), and shaping communication styles, future research can improve both feasibility and acceptability while reducing attrition. For example, we found that dropout was a challenge in the included studies, aligning with previous research [15,78]. Early involvement of target users participating in all stages of future projects, from design to further development and evaluation [77], may be one measure to reduce dropout.

Despite the proliferation of eHealth and mobile health interventions in recent years, especially in populations requiring long-term follow-up and self-management, evidence remains sparse for this specific study population [18]. The benefits regarding efficiency and long-term benefits are minimal [15], and most of the eHealth interventions identified in this review were in the usability testing stage for AYAs with CHD—as underscored by the small number of eligible studies at an early stage of research. The statistical power of individual studies was modest in this review, limiting the generalizability of findings. Risk of bias was high in several studies, limiting confidence in the estimated effects. Interventions were conducted among AYAs with simple, moderate, and complex CHD, making it difficult to draw conclusions that are specific to subgroups, such as those with complex lesions, who may have distinct needs for self-management, education, and support [18,79].

### Research Gaps and Future Directions

The WHO highlights that the only way HCPs can understand and be ready to meet future needs in complex patient care is to collaborate, through an interprofessional focus on education [80]. Future eHealth intervention studies should emphasize interactive communication with AYAs with CHD within interprofessional teams to ensure high-quality long-term care. eHealth holds significant potential for follow-up care. Our findings also highlight the importance of tailoring interventions to disease complexity, particularly for AYAs with higher medical needs, as well as ensuring age-appropriate approaches. This is in line with research in which person-centered care and tailored interventions are supported [76,81].

### Strengths and Limitations

This review has several strengths. The comprehensive search strategy across multiple databases was developed in close cooperation with an experienced research librarian and discussed several times by the core and the entire research group. Furthermore, pairs of researchers assessed eligibility, performed assessments of risk of bias, extracted data, and conducted the narrative synthesis. Another strength was not restricting the literature search by age in the databases, since some of the studies were not indexed as including AYAs. Eligibility based on age was instead assessed during full-text screening.

By restricting the included studies to those explicitly reporting on eHealth interventions involving interaction with HCPs, we may have excluded studies that could have been advantageous to AYAs with CHD. This review was also limited to studies published in specific languages, so relevant studies published in other languages may have been missed. In addition, the included studies were conducted across various countries with differences in health care systems, cultures, educational levels, and economic conditions, contributing to substantial methodological and clinical heterogeneity; findings should therefore be interpreted with caution. Most of the included studies lacked detailed reporting on participants’ backgrounds and socioeconomic status, restricting our ability to assess how these factors may have influenced intervention outcomes or engagement. This limits the interpretation of findings, particularly given known disparities in digital literacy and access to digital technologies and the internet across socioeconomic groups. Finally, substantial heterogeneity in intervention content and focus—including education to improve disease understanding, transition readiness, and self-management—meant that a meta-analysis was neither feasible nor appropriate.

### Conclusion

We found inconsistent evidence regarding the effectiveness of eHealth interventions incorporating HCP interaction for AYAs with CHD. However, such interventions were associated with improvements in disease-specific knowledge, self-efficacy, stress reduction management, physical activity, and psychosocial outcomes. The included studies showed substantial clinical and methodological heterogeneity, an overall high risk of bias, a lack of theoretical foundations, and high dropout rates. Standardized outcome measures and long-term evaluations are needed to clarify the sustained impact and implementation potential of eHealth interventions. Our findings indicate that eHealth interventions involving HCP interaction may have clinical potential for AYAs with CHD in clinical practice, though the current evidence base remains preliminary. Future research is encouraged to include theory foundations, user-centered, and co-designed approaches with AYAs with CHD and HCPs from the initial project phase to enhance relevance, engagement, and long-term sustainability.

## Appendix

Multimedia Appendix 1 PRISMA checklist.

Multimedia Appendix 2 PRISMA-S (PRISMA Literature Search Extension) checklist.

Multimedia Appendix 3 SWiM (Synthesis Without Meta-analysis) checklist.

Multimedia Appendix 4 Literature search strategy.

Multimedia Appendix 5 Characteristics of eHealth interventions studies.

Multimedia Appendix 6 Characteristics of eHealth interventions studies for adolescents and young adults with CHD.

Multimedia Appendix 7 Measured outcomes, instruments, and patient-reported outcomes measures (PROMs) in included studies.

## Abbreviations

AYA: adolescent and young adult
CHD: congenital heart disease
HCP: health care professional
MeSH: Medical Subject Headings
PICO: Population, Intervention, Comparison, and Outcome
PRISMA: Preferred Reporting Items for Systematic Reviews and Meta-Analyses
PRISMA-S: PRISMA Literature Search Extension
RCT: randomized controlled trial
ROB 2: Revised Cochrane risk-of-bias tool for randomized trials
ROBINS-E: Risk of Bias in Non-Randomized Studies of Exposures
SWiM: Synthesis Without Meta-Analysis
WHO: World Health Organization
