# Supplementary material for: Effectiveness of eHealth Interventions for Adolescents and Young Adults With Congenital Heart Disease: Systematic Review
Source: J Med Internet Res. 2026 Jul 8;28:e91424. doi: 10.2196/91424 (PMC13392527; doi:10.2196/91424)
Supplement: Multimedia Appendix 2 [file jmir_v28i1e91424_app2.pdf]

PRISMA-S Checklist

Section/topic	#	Checklist item	Location(s) Reported
**INFORMATION SOURCES AND METHODS**			
Database name	1	Name each individual database searched, stating the platform for each.	Prisma flowchart Abstract Information sources
Multi-database searching	2	If databases were searched simultaneously on a single platform, state the name of the platform, listing all of the databases searched.	1)
Study registries	3	List any study registries searched.	Prisma flowchart
Online resources and browsing	4	Describe any online or print source purposefully searched or browsed (e.g., tables of contents, print conference proceedings, web sites), and how this was done.	2)
Citation searching	5	Indicate whether cited references or citing references were examined, and describe any methods used for locating cited/citing references (e.g., browsing reference lists, using a citation index, setting up email alerts for references citing included studies).	Information sources
Contacts	6	Indicate whether additional studies or data were sought by contacting authors, experts, manufacturers, or others.	3)
Other methods	7	Describe any additional information sources or search methods used.	4)
**SEARCH STRATEGIES**			
Full search strategies	8	Include the search strategies for each database and information source, copied and pasted exactly as run.	Supplementary file X
Limits and restrictions	9	Specify that no limits were used, or describe any limits or restrictions applied to a search (e.g., date or time period, language, study design) and provide justification for their use.	Search strategy
Search filters	10	Indicate whether published search filters were used (as originally designed or modified), and if so, cite the filter(s) used.	Search strategy

Prior work	11	Indicate when search strategies from other literature reviews were adapted or reused for a substantive part or all of the search, citing the previous review(s).	5)
Updates	12	Report the methods used to update the search(es) (e.g., rerunning searches, email alerts).	Information sources
Dates of searches	13	For each search strategy, provide the date when the last search occurred.	Abstract, Information sources
**PEER REVIEW**			
Peer review	14	Describe any search peer review process.	Search strategy
**MANAGING RECORDS**			
Total Records	15	Document the total number of records identified from each database and other information sources.	Prisma flowchart
Deduplication	16	Describe the processes and any software used to deduplicate records from multiple database searches and other information sources.	Selection process

PRISMA-S: An Extension to the PRISMA Statement for Reporting Literature Searches in Systematic Reviews
Rethlefsen ML, Kirtley S, Waffenschmidt S, Ayala AP, Moher D, Page MJ, Koffel JB, PRISMA-S Group.
Last updated February 27, 2020.

1) Databases were not searched simultaneously on a single platform
2) No online or print source was purposefully searched or browsed (e.g., tables of contents, print conference proceedings, web sites)
3) No authors, experts, manufacturers, or others were contacted to identify additional studies
4) No additional information sources or search methods were used.
5) The search strategy was built by using MeSH-terms and examining “used-for” terms in the MeSH-thesaurus, in addition to examining the search results to broaden the search
