# Supplementary material for: Effectiveness of eHealth Interventions for Adolescents and Young Adults With Congenital Heart Disease: Systematic Review
Source: J Med Internet Res. 2026 Jul 8;28:e91424. doi: 10.2196/91424 (PMC13392527; doi:10.2196/91424)
Supplement: Multimedia Appendix 4 [file jmir_v28i1e91424_app4.pdf]

Literature search strategy

1	exp Heart Defects, Congenital/
2	(heart* adj2 (abnormal* or anomal* or malform*)).tw,kf.
3	(congenital adj2 (heart* or valve* or mitral* or aort* or tricuspid) adj2 (defect* or disease* or abnormal* or anomal* or malform*)).tw,kf.
4	(Syndrome* adj2 (22q11 or "22q11.2" or "Pharyngeal Pouch" or "Autosomal Dominant Opitz G-Bbb" or "Thymic Aplasia*" or "Thymic Hypoplasia*" or Velocardiofacial or "Velo Cardio Facial" or VCF or Sedlackova* or Shprintzen* or "Conotruncal Anomaly Face" or CTAF or Alagille* or Cardiovertebral or Hepatofacioneurocardiovertebral or "Watson Miller*" or Barth* or "Bland White Garland" or Kartagener* or Siewert* or Eisenmenger* or Lutembacher* or Heterotaxy or Polysplenia* or Asplenia* or Ivemark* or "Left Heart" or LEOPARD* or "Cardio-Cutaneous" or Lentigine* or Noonan* or "Long QT" or Andersen* or "Lange Nielsen*" or Jervell* or "Cardio Auditory Syncope" or "Surdo Cardiac" or "Romano Ward*" or "Ward Romano*" or Marfan* or Turner* or Patau* or "Chromosome 13" or "Trisomy 13" or "Trisomy 18" or "Trisomy E" or Edward* or "Bonnevie Ullrich*" or "Wolf* Parkinson White*" or "Auriculoventricular Accessory Pathway" or WPW or "Anomalous Ventricular Excitation" or "False Bundle-Branch Block")).tw,kf.
5	(deletion adj2 (22q11 or "22q11.2")).tw,kf.
6	(CHD or CHDs or CCHD or CCHDs or "22q11.2DS" or DiGeorge* or "velofacial hypoplasia" or ALCAPA or ARVD-C or "3-Methylglutaconicaciduria" or "3-Methylglutaconic Aciduria" or "cor Triatriatum" or Dextrocardi* or dextroversion or "dextro rotation" or dextrorotation or dextroposition or "Ciliary Dyskinesi*" or Levocardia or Laevocardia or "univentricular heart*" or "monoventricular heart*" or "single ventric*" or "Truncus Arterios*" or "Interrupted aortic arch*" or "ventricular inversion*").tw,kf.
7	(cyanotic adj2 (defect* or disease*)).tw,kf.
8	((ebstein* or "Taussig Bing*") adj3 (sequence* or anomal* or malform* or abnorm* or disease)).tw,kf.
9	((Anomal* or abnormal*) adj2 (coronar* or aortic or aorta* or aorto* or venous)).tw,kf.
10	(dysplasia adj2 (Arteriohepatic or Ventricular)).tw,kf.
11	((aortic or aorta or aorto) adj3 (Coarctation* or fistula*)).tw,kf.
12	((Aortoventricular or Aorticoventricular or "Aortic* Ventricular" or "Aorto* Ventricular") adj2 tunnel*).tw,kf.
13	((ductular or "left heart*") adj2 hypoplas*).tw,kf.
14	((Bicuspid or Quadricuspid) adj2 (valve* or aortic)).tw,kf.
15	(atrium* adj2 subdivided).tw,kf.
16	(myocardial adj2 (bridge* or bridging*)).tw,kf.
17	((crisscross or "criss cross" or Triatrial) adj2 heart*).tw,kf.
18	(Kartagener* adj2 triad*).tw,kf.

19	(Bronchiectas* adj2 Polynesian).tw,kf.
20	((paten* or ligat* or occlude* or persisten* or obliteration) adj3 ("ductus arterios*" or "truncus arterios*" or oval* or "ductus botalli")).tw,kf.
21	(((Cordis or heart* or cardiac) adj1 Ectop*) or ectocardia or exocardia).tw,kf.
22	(Eisenmenger* adj1 (complex or disease or tetralogy)).tw,kf.
23	((heart or cardiac or aortopulmonary or aorticopulmonary or atrial or atrium or interatrial or interauricular or ventricle or ventricular or intraventricular or interventricular or atrioventricular) adj2 (septal or septum) adj2 defect*).tw,kf.
24	((endocardial or atrioventricular) adj2 Cushion adj2 Defect*).tw,kf.
25	(AVSD or "ostium primum ASD*" or "unrepaired secundum ASD*" or VSD or "persistent ostium secundum" or "patent ostium secundum").tw,kf.
26	(("double-outlet" or single) adj1 (right or left) adj3 ventric*).tw,kf.
27	(Heterotax* adj2 visceral).tw,kf.
28	(situs adj2 ambigu*).tw,kf.
29	("atrial isomerism*" adj2 (left or right)).tw,kf.
30	((tetralogy or trilogy) adj2 fallot*).tw,kf.
31	((transposition* adj3 ("great arter*" or "great vessel*" or "large vessel*")) or (Dextrotransposition* or D-TGA* or Dextro-TGA* or "dexto-transposition*" or "d-transposition*" or "dextro-looped TGA*" or CCTGA* or CC-TGA* or Levotransposition* or "Levo-transposition*" or "L-looped transposition*or Levo-TGA*" or "L-TGA*")).tw,kf.
32	((Mitral or tricuspid or pulmonary or "right atrioventricular" or "right av") adj3 atresi*).tw,kf.
33	(congenital and "ventricular outflow obstruction*").tw,kf.
34	(("Pulmonary valve*" or "Peripheral pulmonary" or Subvalvar or Supravalvar or subvalvular or supravalvular or subaortic) adj3 (regurgitation* or stenos*)).tw,kf.
35	(sinus adj2 (Valsalva or aort* or veno*) adj3 (fistula* or aneurysm* or aneurism* or defect*)).tw,kf.
36	(straddling adj2 "atrioventricular valve*").tw,kf.
37	((heart or cardiac or intercardiac or intracardiac or intercardial or interventricular or ventriculoatrial or atrioventricular or left or right) adj3 shunt).tw,kf.
38	((Common or Persistent) adj2 "atrioventricular canal").tw,kf.
39	("Ventricular noncompaction" or "Ventricular non-compaction" or INVM or LVNC).tw,kf.
40	1 or 2 or 3 or 4 or 5 or 6 or 7 or 8 or 9 or 10 or 11 or 12 or 13 or 14 or 15 or 16 or 17 or 18 or 19 or 20 or 21 or 22 or 23 or 24 or 25 or 26 or 27 or 28 or 29 or 30 or 31 or 32 or 33 or 34 or 35 or 36 or 37 or 38 or 39
41	exp Telemedicine/
42	("e-health*" or ehealth* or "m-health*" or mhealth* or telehealth* or "tele-health*" or

	((mobile or remote or digital or online or virtual) adj2 health*)).tw,kf.
43	(e-medic* or emedic* or telemedic* or tele-medic* or "virtual medic*" or "digital medicine" or "online medicine").tw,kf.
44	(telecardiology or "tele-cardiology").tw,kf.
45	(telecare* or "tele-care*" or "e-care*" or ehomecare* or "e-homecare*" or "e-home-care*" or ((remote or virtual or digital) adj (care* or homecare* or "home-care*"))).tw,kf.
46	(telenursing or "tele-nursing" or ((virtual or digital or online or remote) adj2 nursing)).tw,kf.
47	(teleconsultation* or "tele-consultation*" or cyberconsultation* or "cyber-consultation*" or "e-consultation*" or econsultation* or videoconsultation* or ((remote or electronic or email or internet or online or web or webbased or video) adj2 consultation*)).tw,kf.
48	(telemonitor* or "tele-monitor*" or "tele-surveillance" or telesurveillance or ((distant or remote or virtual* or electronic or digital*) adj2 (monitor* or surveillance))).tw,kf.
49	(telerehabilitation* or "tele-rehabilitation*" or "e-rehabilitation*" or ((remote or electronic or digital or virtual or email or internet or online or web or webbased or video) adj2 rehabilitation*)).tw,kf.
50	(tele-psychiatry or telepsychiatry or telepsychology or tele-psychology or teletherapy or tele-therapy or ((remote or electronic or virtual or "email-based" or email or internet or "internet-based" or online or web or "web-based" or webbased or video) adj (therapy or psychiatry or psychology))).tw,kf.
51	Internet-Based Intervention/
52	("web-based intervention*" or "webbased intervention*" or "internet-based intervention*" or "internet intervention*" or "online-based intervention*" or "online-intervention*" or "web intervention*" or "digital intervention*" or "digital-based intervention*").tw,kf.
53	((treatment adj3 internet) or ((digital or "digital-based" or online or "online-based") adj treatment)).tw,kf.
54	(virtual adj2 (hospital* or ward*)).tw,kf.
55	41 or 42 or 43 or 44 or 45 or 46 or 47 or 48 or 49 or 50 or 51 or 52 or 53 or 54
56	computers, handheld/
57	cell phone/ or smartphone/
58	wearable electronic devices/ or fitness trackers/ or smart glasses/
59	("cell-phone*" or cellphone* or smartphone* or "smart-phone*" or ((cellular or mobile) adj (phone* or telephone*))).tw,kf.
60	(ipad or "mobile device*" or "palm-pilot*" or palmpilot* or "personal digital assistant*" or touchscreen* or touch-screen* or ((handheld or "hand-held" or palm* or pda or tablet or pocket) adj1 computer*)).tw,kf.
61	(smarthome* or "smart-home*").tw,kf.
62	(((wearable or wireless) adj2 (technolog* or electronic* or device*)) or "fitness tracker*" or "activity tracker*" or "smart glass*" or pedometer* or smartwatch* or "smart-watch*" or healthwatch* or "health-watch*").tw,kf.

63	56 or 57 or 58 or 59 or 60 or 61 or 62
64	Wireless Technology/
65	(wireless adj1 communication*).tw,kf.
66	exp Telemetry/
67	(telemetr* or "remote sensing").tw,kf.
68	telecommunications/ or electronic mail/ or text messaging/ or videoconferencing/
69	(telecommunication* or tele-conferenc* or teleconferenc* or videoconferenc* or "video-conferenc*" or "text messag*" or textmessag* or texting or "short message service*" or sms or "electronic mail*" or email* or "e-mail*").tw,kf.
70	internet/ or "internet use"/
71	"Cell Phone Use"/
72	social media/ or "social medi*".tw,kf.
73	Mobile Applications/
74	gamification/ or gamification.tw,kf.
75	exp virtual reality/ or ("virtual reality" or avatar*).tw,kf.
76	(app or apps or "app-based" or (application* adj2 (mobile or telephone* or web or internet or tablet))).tw,kf.
77	(((digital or virtual or robot*) adj2 (assistant*1 or companion*)) or (robot* adj2 social*)).tw,kf.
78	64 or 65 or 66 or 67 or 68 or 69 or 70 or 71 or 72 or 73 or 74 or 75 or 76 or 77
79	55 or 63 or 78
80	40 and 79
81	limit 80 to yr="2010 -Current"
82	limit 81 to (danish or english or norwegian or spanish or swedish)
83	limit 82 to (comment or congress or editorial or letter)
84	82 not 83

EMBASE

1	exp congenital heart disease/
2	(heart* adj2 (abnormal* or anomal* or malform*)).tw,kf.
3	(congenital adj2 (heart* or valve* or mitral* or aort* or tricuspid) adj2 (defect* or disease* or abnormal* or anomal* or malform*)).tw,kf.
4	(Syndrome* adj2 (22q11 or "22q11.2" or "Pharyngeal Pouch" or "Autosomal Dominant Opitz G-Bbb" or "Thymic Aplasia*" or "Thymic Hypoplasia*" or Velocardiofacial or "Velo Cardio Facial" or VCF or Sedlackova* or Shprintzen* or "Conotruncal Anomaly Face" or CTAF or Alagille* or Cardiovertebral or Hepatofacioneurocardiovertebral or "Watson Miller*" or Barth* or "Bland White Garland" or Kartagener* or Siewert* or Eisenmenger* or Lutembacher* or Heterotaxy or Polysplenia* or Asplenia* or Ivemark* or "Left Heart" or LEOPARD*

	or "Cardio-Cutaneous" or Lentigine* or Noonan* or "Long QT" or Andersen* or "Lange Nielsen*" or Jervell* or "Cardio Auditory Syncope" or "Surdo Cardiac" or "Romano Ward*" or "Ward Romano*" or Marfan* or Turner* or Patau* or "Chromosome 13" or "Trisomy 13" or "Trisomy 18" or "Trisomy E" or Edward* or "Bonnevie Ullrich*" or "Wolf* Parkinson White*" or "Auriculoventricular Accessory Pathway" or WPW or "Anomalous Ventricular Excitation" or "False Bundle-Branch Block")).tw,kf.
5	(deletion adj2 (22q11 or "22q11.2")).tw,kf.
6	(CHD or CHDs or CCHD or CCHDs or "22q11.2DS" or DiGeorge* or "velofacial hypoplasia" or ALCAPA or ARVD-C or "3-Methylglutaconicaciduria" or "3-Methylglutaconic Aciduria" or "cor Triatriatum" or Dextrocardi* or dextroversion or "dextro rotation" or dextrorotation or dextroposition or "Ciliary Dyskinesi*" or Levocardia or Laevocardia or "univentricular heart*" or "monoventricular heart*" or "single ventric*" or "Truncus Arterios*" or "Interrupted aortic arch*" or "ventricular inversion*").tw,kf.
7	(cyanotic adj2 (defect* or disease*)).tw,kf.
8	((ebstein* or "Taussig Bing*") adj3 (sequence* or anomal* or malform* or abnorm* or disease)).tw,kf.
9	((Anomal* or abnormal*) adj2 (coronar* or aortic or aorta* or aorto* or venous)).tw,kf.
10	(dysplasia adj2 (Arteriohepatic or Ventricular)).tw,kf.
11	((aortic or aorta or aorto) adj3 (Coarctation* or fistula*)).tw,kf.
12	((Aortoventricular or Aorticoventricular or "Aortic* Ventricular" or "Aorto* Ventricular") adj2 tunnel*).tw,kf.
13	((ductular or "left heart*") adj2 hypoplas*).tw,kf.
14	((Bicuspid or Quadricuspid) adj2 (valve* or aortic)).tw,kf.
15	(atrium* adj2 subdivided).tw,kf.
16	(myocardial adj2 (bridge* or bridging*)).tw,kf.
17	((crisscross or "criss cross" or Triatrial) adj2 heart*).tw,kf.
18	(Kartagener* adj2 triad*).tw,kf.
19	(Bronchiectas* adj2 Polynesian).tw,kf.
20	((paten* or ligat* or occlude* or persisten* or obliteration) adj3 ("ductus arterios*" or "truncus arterios*" or oval* or "ductus botalli")).tw,kf.
21	(((Cordis or heart* or cardiac) adj1 Ectop*) or ectocardia or exocardia).tw,kf.
22	(Eisenmenger* adj1 (complex or disease or tetralogy)).tw,kf.
23	((heart or cardiac or aortopulmonary or aorticopulmonary or atrial or atrium or interatrial or interauricular or ventricle or ventricular or intraventricular or interventricular or atrioventricular) adj2 (septal or septum) adj2 defect*).tw,kf.
24	((endocardial or atrioventricular) adj2 Cushion adj2 Defect*).tw,kf.
25	(AVSD or "ostium primum ASD*" or "unrepaired secundum ASD*" or VSD or "persistent ostium secundum" or "patent ostium secundum").tw,kf.
26	(("double-outlet" or single) adj1 (right or left) adj3 ventric*).tw,kf.
27	(Heterotax* adj2 visceral).tw,kf.
28	(situs adj2 ambigu*).tw,kf.
29	("atrial isomerism*" adj2 (left or right)).tw,kf.
30	((tetralogy or trilogy) adj2 fallot*).tw,kf.
31	((transposition* adj3 ("great arter*" or "great vessel*" or "large vessel*")) or (Dextrotransposition* or D-TGA* or Dextro-TGA* or "dexto-transposition*" or "d-transposition*" or "dextro-looped TGA*" or CCTGA* or CC-TGA* or

	Levotransposition* or "Levo-transposition*" or "L-looped transposition*or Levo-TGA*" or "L-TGA*")).tw,kf.
32	((Mitral or tricuspid or pulmonary or "right atrioventricular" or "right av") adj3 atresi*).tw,kf.
33	(congenital and "ventricular outflow obstruction*").tw,kf.
34	(("Pulmonary valve*" or "Peripheral pulmonary" or Subvalvar or Supravalvar or subvalvular or supravalvular or subaortic) adj3 (regurgitation* or stenos*)).tw,kf.
35	(sinus adj2 (Valsalva or aort* or veno*) adj3 (fistula* or aneurysm* or aneurism* or defect*)).tw,kf.
36	(straddling adj2 "atrioventricular valve*").tw,kf.
37	((heart or cardiac or intercardiac or intracardiac or intercardial or interventricular or ventriculoatrial or atrioventricular or left or right) adj3 shunt).tw,kf.
38	((Common or Persistent) adj2 "atrioventricular canal").tw,kf.
39	("Ventricular noncompaction" or "Ventricular non-compaction" or INVM or LVNC).tw,kf.
40	1 or 2 or 3 or 4 or 5 or 6 or 7 or 8 or 9 or 10 or 11 or 12 or 13 or 14 or 15 or 16 or 17 or 18 or 19 or 20 or 21 or 22 or 23 or 24 or 25 or 26 or 27 or 28 or 29 or 30 or 31 or 32 or 33 or 34 or 35 or 36 or 37 or 38 or 39
41	exp telehealth/
42	("e-health*" or ehealth* or "m-health*" or mhealth* or telehealth* or "tele-health*" or ((mobile or remote or digital or online or virtual) adj2 health*)).tw,kf.
43	(e-medic* or emedic* or telemedic* or tele-medic* or "virtual medic*" or "digital medicine" or "online medicine").tw,kf.
44	(telecardiology or "tele-cardiology").tw,kf.
45	(telecare* or "tele-care*" or "e-care*" or ehomecare* or "e-homecare*" or "e-home-care*" or ((remote or virtual or digital) adj (care* or homecare* or "home-care*"))).tw,kf.
46	(telenursing or "tele-nursing" or ((virtual or digital or online or remote) adj2 nursing)).tw,kf.
47	(teleconsultation* or "tele-consultation*" or cyberconsultation* or "cyber-consultation*" or "e-consultation*" or econsultation* or videoconsultation* or ((remote or electronic or email or internet or online or web or webbased or video) adj2 consultation*)).tw,kf.
48	(telemonitor* or "tele-monitor*" or "tele-surveillance" or telesurveillance or ((distant or remote or virtual* or electronic or digital*) adj2 (monitor* or surveillance))).tw,kf.
49	(telerehabilitation* or "tele-rehabilitation*" or "e-rehabilitation*" or ((remote or electronic or digital or virtual or email or internet or online or web or webbased or video) adj2 rehabilitation*)).tw,kf.
50	(tele-psychiatry or telepsychiatry or telepsychology or tele-psychology or teletherapy or tele-therapy or ((remote or electronic or virtual or "email-based" or email or internet or "internet-based" or online or web or "web-based" or webbased or video) adj (therapy or psychiatry or psychology))).tw,kf.
51	web-based intervention/
52	("web-based intervention*" or "webbased intervention*" or "internet-based intervention*" or "internet intervention*" or "online-based intervention*" or "online-intervention*" or "web intervention*" or "digital intervention*" or "digital-based intervention*").tw,kf.
53	((treatment adj3 internet) or ((digital or "digital-based" or online or "online-based")

	adj treatment)).tw,kf.
54	(virtual adj2 (hospital* or ward*)).tw,kf.
55	41 or 42 or 43 or 44 or 45 or 46 or 47 or 48 or 49 or 50 or 51 or 52 or 53 or 54
56	exp personal computer/
57	personal digital assistant/
58	exp mobile phone/
59	("cell-phone*" or cellphone* or smartphone* or "smart-phone*" or ((cellular or mobile) adj (phone* or telephone*))).tw,kf.
60	(ipad or "mobile device*" or "palm-pilot*" or palmpilot* or "personal digital assistant*" or touchscreen* or touch-screen* or ((handheld or "hand-held" or palm* or pda or tablet or pocket) adj1 computer*)).tw,kf.
61	(smarthome* or "smart-home*").tw,kf.
62	(((wearable or wireless) adj2 (technolog* or electronic* or device*)) or "fitness tracker*" or "activity tracker*" or "smart glass*" or pedometer* or smartwatch* or "smart-watch*" or healthwatch* or "health-watch*").tw,kf.
63	56 or 57 or 58 or 59 or 60 or 61 or 62
64	wireless communication/
65	telecommunication/ or teleconference/
66	(wireless adj1 communication*).tw,kf.
67	exp telemetry/
68	(telemetr* or "remote sensing").tw,kf.
69	e-mail/ or text messaging/ or videoconferencing/
70	(telecommunication* or tele-conferenc* or teleconferenc* or videoconferenc* or "video-conferenc*" or "text messag*" or textmessag* or texting or "short message service*" or sms or "electronic mail*" or email* or "e-mail*").tw,kf.
71	exp interactive voice response system/
72	*Internet/ or *"internet use"/
73	"cell phone use"/
74	*social media/ or "social medi*".tw,kf.
75	exp mobile application/
76	gamification/ or gamification.tw,kf.
77	virtual reality/ or ("virtual reality" or avatar*).tw,kf.
78	(app or apps or "app-based" or (application* adj2 (mobile or telephone* or web or internet or tablet))).tw,kf.
79	(((digital or virtual or robot*) adj2 (assistant*1 or companion*)) or (robot* adj2 social*)).tw,kf.
80	64 or 65 or 66 or 67 or 68 or 69 or 70 or 71 or 72 or 73 or 74 or 75 or 76 or 77 or 78 or 79
81	55 or 63 or 80
82	40 and 81
83	limit 82 to yr="2010 -Current"
84	limit 83 to (danish or english or norwegian or spanish or swedish)
85	limit 84 to (conference abstracts or conference paper or "conference review" or editorial or letter)
86	84 not 85

PsycINFO

1	exp heart disorders/

2	(heart* adj2 (abnormal* or anomal* or malform*)).tw.
3	(congenital adj2 (heart* or valve* or mitral* or aort* or tricuspid) adj2 (defect* or disease* or abnormal* or anomal* or malform*)).tw.
4	(Syndrome* adj2 (22q11 or "22q11.2" or "Pharyngeal Pouch" or "Autosomal Dominant Opitz G-Bbb" or "Thymic Aplasia*" or "Thymic Hypoplasia*" or Velocardiofacial or "Velo Cardio Facial" or VCF or Sedlackova* or Shprintzen* or "Conotruncal Anomaly Face" or CTAF or Alagille* or Cardiovertebral or Hepatofacioneurocardiovertebral or "Watson Miller*" or Barth* or "Bland White Garland" or Kartagener* or Siewert* or Eisenmenger* or Lutembacher* or Heterotaxy or Polysplenia* or Asplenia* or Ivemark* or "Left Heart" or LEOPARD* or "Cardio-Cutaneous" or Lentigine* or Noonan* or "Long QT" or Andersen* or "Lange Nielsen*" or Jervell* or "Cardio Auditory Syncope" or "Surdo Cardiac" or "Romano Ward*" or "Ward Romano*" or Marfan* or Turner* or Patau* or "Chromosome 13" or "Trisomy 13" or "Trisomy 18" or "Trisomy E" or Edward* or "Bonnevie Ullrich*" or "Wolf* Parkinson White*" or "Auriculoventricular Accessory Pathway" or WPW or "Anomalous Ventricular Excitation" or "False Bundle-Branch Block")).tw.
5	(deletion adj2 (22q11 or "22q11.2")).tw.
6	(CHD or CHDs or CCHD or CCHDs or "22q11.2DS" or DiGeorge* or "velofacial hypoplasia" or ALCAPA or ARVD-C or "3-Methylglutaconicaciduria" or "3-Methylglutaconic Aciduria" or "cor Triatriatum" or Dextrocardi* or dextroversion or "dextro rotation" or dextrorotation or dextroposition or "Ciliary Dyskinesi*" or Levocardia or Laevocardia or "univentricular heart*" or "monoventricular heart*" or "single ventric*" or "Truncus Arterios*" or "Interrupted aortic arch*" or "ventricular inversion*").tw.
7	(cyanotic adj2 (defect* or disease*)).tw.
8	((ebstein* or "Taussig Bing*") adj3 (sequence* or anomal* or malform* or abnorm* or disease)).tw.
9	((Anomal* or abnormal*) adj2 (coronar* or aortic or aorta* or aorto* or venous)).tw.
10	(dysplasia adj2 (Arteriohepatic or Ventricular)).tw.
11	((aortic or aorta or aorto) adj3 (Coarctation* or fistula*)).tw.
12	((Aortoventricular or Aorticoventricular or "Aortic* Ventricular" or "Aorto* Ventricular") adj2 tunnel*).tw.
13	((ductular or "left heart*") adj2 hypoplas*).tw.
14	((Bicuspid or Quadricuspid) adj2 (valve* or aortic)).tw.
15	(atrium* adj2 subdivided).tw.
16	(myocardial adj2 (bridge* or bridging*)).tw.
17	((crisscross or "criss cross" or Triatrial) adj2 heart*).tw.
18	(Kartagener* adj2 triad*).tw.
19	(Bronchiectas* adj2 Polynesian).tw.
20	((paten* or ligat* or occlude* or persisten* or obliteration) adj3 ("ductus arterios*" or "truncus arterios*" or oval* or "ductus botalli")).tw.

21	(((Cordis or heart* or cardiac) adj1 Ectop*) or ectocardia or exocardia).tw.
22	(Eisenmenger* adj1 (complex or disease or tetralogy)).tw.
23	((heart or cardiac or aortopulmonary or aorticopulmonary or atrial or atrium or interatrial or interauricular or ventricle or ventricular or intraventricular or interventricular or atrioventricular) adj2 (septal or septum) adj2 defect*).tw.
24	((endocardial or atrioventricular) adj2 Cushion adj2 Defect*).tw.
25	(AVSD or "ostium primum ASD*" or "unrepaired secundum ASD*" or VSD or "persistent ostium secundum" or "patent ostium secundum").tw.
26	(("double-outlet" or single) adj1 (right or left) adj3 ventric*).tw.
27	(Heterotax* adj2 visceral).tw.
28	(situs adj2 ambigu*).tw.
29	("atrial isomerism*" adj2 (left or right)).tw.
30	((tetralogy or trilogy) adj2 fallot*).tw.
31	((transposition* adj3 ("great arter*" or "great vessel*" or "large vessel*")) or (Dextrotransposition* or D-TGA* or Dextro-TGA* or "dexto-transposition*" or "d-transposition*" or "dextro-looped TGA*" or CCTGA* or CC-TGA* or Levotransposition* or "Levo-transposition*" or "L-looped transposition*or Levo-TGA*" or "L-TGA*")).tw.
32	((Mitral or tricuspid or pulmonary or "right atrioventricular" or "right av") adj3 atresi*).tw.
33	(congenital and "ventricular outflow obstruction*").tw.
34	(("Pulmonary valve*" or "Peripheral pulmonary" or Subvalvar or Supravalvar or subvalvular or supravalvular or subaortic) adj3 (regurgitation* or stenos*)).tw.
35	(sinus adj2 (Valsalva or aort* or veno*) adj3 (fistula* or aneurysm* or aneurism* or defect*)).tw.
36	(straddling adj2 "atrioventricular valve*").tw.
37	((heart or cardiac or intercardiac or intracardiac or intercardial or interventricular or ventriculoatrial or atrioventricular or left or right) adj3 shunt).tw.
38	((Common or Persistent) adj2 "atrioventricular canal").tw.
39	("Ventricular noncompaction" or "Ventricular non-compaction" or INVM or LVNC).tw.
40	1 or 2 or 3 or 4 or 5 or 6 or 7 or 8 or 9 or 10 or 11 or 12 or 13 or 14 or 15 or 16 or 17 or 18 or 19 or 20 or 21 or 22 or 23 or 24 or 25 or 26 or 27 or 28 or 29 or 30 or 31 or 32 or 33 or 34 or 35 or 36 or 37 or 38 or 39
41	exp electronic health services/
42	("e-health*" or ehealth* or "m-health*" or mhealth* or telehealth* or "tele-health*" or ((mobile or remote or digital or online or virtual) adj2 health*)).tw.
43	(e-medic* or emedic* or telemedic* or tele-medic* or "virtual medic*" or "digital medicine" or "online medicine").tw.
44	(telecardiology or "tele-cardiology").tw.

45	(telecare* or "tele-care*" or "e-care*" or ehomecare* or "e-homecare*" or "e-home-care*" or ((remote or virtual or digital) adj (care* or homecare* or "home-care*"))).tw.
46	(telenursing or "tele-nursing" or ((virtual or digital or online or remote) adj2 nursing)).tw.
47	(teleconsultation* or "tele-consultation*" or cyberconsultation* or "cyber-consultation*" or "e-consultation*" or econsultation* or videoconsultation* or ((remote or electronic or email or internet or online or web or webbased or video) adj2 consultation*)).tw.
48	(telemonitor* or "tele-monitor*" or "tele-surveillance" or telesurveillance or ((distant or remote or virtual* or electronic or digital*) adj2 (monitor* or surveillance))).tw.
49	(telerehabilitation* or "tele-rehabilitation*" or "e-rehabilitation*" or ((remote or electronic or digital or virtual or email or internet or online or web or webbased or video) adj2 rehabilitation*)).tw.
50	(tele-psychiatry or telepsychiatry or telepsychology or tele-psychology or teletherapy or tele-therapy or ((remote or electronic or virtual or "email-based" or email or internet or "internet-based" or online or web or "web-based" or webbased or video) adj (therapy or psychiatry or psychology))).tw.
51	("web-based intervention*" or "webbased intervention*" or "internet-based intervention*" or "internet intervention*" or "online-based intervention*" or "online-intervention*" or "web intervention*" or "digital intervention*" or "digital-based intervention*").tw.
52	((treatment adj3 internet) or ((digital or "digital-based" or online or "online-based") adj treatment)).tw.
53	(virtual adj2 (hospital* or ward*)).tw.
54	41 or 42 or 43 or 44 or 45 or 46 or 47 or 48 or 49 or 50 or 51 or 52 or 53
55	exp microcomputers/
56	exp mobile devices/
57	wearable devices/
58	("cell-phone*" or cellphone* or smartphone* or "smart-phone*" or ((cellular or mobile) adj (phone* or telephone*))).tw.
59	(ipad or "mobile device*" or "palm-pilot*" or palmpilot* or "personal digital assistant*" or touchscreen* or touch-screen* or ((handheld or "hand-held" or palm* or pda or tablet or pocket) adj1 computer*)).tw.
60	(smarthome* or "smart-home*").tw.
61	(((wearable or wireless) adj2 (technolog* or electronic* or device*)) or "fitness tracker*" or "activity tracker*" or "smart glass*" or pedometer* or smartwatch* or "smart-watch*" or healthwatch* or "health-watch*").tw.
62	55 or 57 or 58 or 59 or 60 or 61
63	exp wireless technologies/
64	(wireless adj1 communication*).tw.
65	(telemetr* or "remote sensing").tw.
66	telecommunications media/ or teleconferencing/ or videoconferencing/ or computer mediated communication/ or electronic communication/ or text messaging/

67	(telecommunication* or tele-conferenc* or teleconferenc* or videoconferenc* or "video-conferenc*" or "text messag*" or textmessag* or texting or "short message service*" or sms or "electronic mail*" or email* or "e-mail*").tw.
68	internet/ or internet usage/
69	mobile phones/ or "smartphone use"/
70	exp social media/ or "social medi*".tw.
71	mobile applications/
72	gamification.tw.
73	exp virtual reality/ or virtual environment/ or avatars/ or ("virtual reality" or avatar*).tw.
74	(app or apps or "app-based" or (application* adj2 (mobile or telephone* or web or internet or tablet))).tw.
75	(((digital or virtual or robot*) adj2 (assistant*1 or companion*)) or (robot* adj2 social*)).tw.
76	63 or 64 or 65 or 66 or 67 or 68 or 69 or 70 or 71 or 72 or 73 or 74 or 75
77	54 or 62 or 76
78	40 and 77
79	limit 78 to yr="2010 -Current"
80	limit 79 to (danish or english or norwegian or spanish or swedish)
81	limit 80 to (conference proceedings or "handbook/manual" or reference book or "textbook/study guide" or "comment/reply" or editorial or letter)
82	80 not 81

CINAHL

S1	(MH "Heart Defects, Congenital+")	29759
S2	TI (heart* N1 (abnormal* or anomal* or malform*)) or AB (heart* N1 (abnormal* or anomal* or malform*))	1156
S3	TI (congenital N1 (heart* or valve* or mitral* or aort* or tricuspid) N1 (defect* or disease* or abnormal* or anomal* or malform*)) or AB (congenital N1 (heart* or valve* or mitral* or aort* or tricuspid) N1 (defect* or disease* or abnormal* or anomal* or malform*))	10267
S4	TI (Syndrome* N1 (22q11 or "22q11.2" or "Pharyngeal Pouch" or "Autosomal Dominant Opitz G-Bbb" or "Thymic Aplasia*" or "Thymic Hypoplasia*" or Velocardiofacial or "Velo Cardio Facial" or VCF or Sedlackova* or Shprintzen* or "Conotruncal Anomaly Face" or CTAF or Alagille* or Cardiovertebral or Hepatofacioneurocardiovertebral or "Watson Miller*" or Barth* or "Bland White Garland" or Kartagener* or Siewert* or Eisenmenger* or Lutembacher* or Heterotaxy or Polysplenia* or Asplenia* or Ivemark* or "Left Heart" or LEOPARD* or "Cardio-Cutaneous" or Lentigine* or Noonan* or "Long QT" or Andersen* or "Lange Nielsen*" or Jervell* or "Cardio Auditory Syncope" or "Surdo Cardiac" or "Romano Ward*" or "Ward	7562

	Romano*" or Marfan* or Turner* or Patau* or "Chromosome 13" or "Trisomy 13" or "Trisomy 18" or "Trisomy E" or Edward* or "Bonnevie Ullrich*" or "Wolf* Parkinson White*" or "Auriculoventricular Accessory Pathway" or WPW or "Anomalous Ventricular Excitation" or "False Bundle-Branch Block")) or<br>AB (Syndrome* N1 (22q11 or "22q11.2" or "Pharyngeal Pouch" or "Autosomal Dominant Opitz G-Bbb" or "Thymic Aplasia*" or "Thymic Hypoplasia*" or Velocardiofacial or "Velo Cardio Facial" or VCF or Sedlackova* or Shprintzen* or "Conotruncal Anomaly Face" or CTAF or Alagille* or Cardiovertebral or Hepatofacioneurocardiovertebral or "Watson Miller*" or Barth* or "Bland White Garland" or Kartagener* or Siewert* or Eisenmenger* or Lutembacher* or Heterotaxy or Polysplenia* or Asplenia* or Ivemark* or "Left Heart" or LEOPARD* or "Cardio-Cutaneous" or Lentigine* or Noonan* or "Long QT" or Andersen* or "Lange Nielsen*" or Jervell* or "Cardio Auditory Syncope" or "Surdo Cardiac" or "Romano Ward*" or "Ward Romano*" or Marfan* or Turner* or Patau* or "Chromosome 13" or "Trisomy 13" or "Trisomy 18" or "Trisomy E" or Edward* or "Bonnevie Ullrich*" or "Wolf* Parkinson White*" or "Auriculoventricular Accessory Pathway" or WPW or "Anomalous Ventricular Excitation" or "False Bundle-Branch Block"))	
S5	TI (deletion N1 (22q11 or "22q11.2")) or<br>AB (deletion N1 (22q11 or "22q11.2"))	621
S6	TI (CHD or CHDs or CCHD or CCHDs or "22q11.2DS" or DiGeorge* or "velofacial hypoplasia" or ALCAPA or ARVD-C or "3-Methylglutaconicaciduria" or "3-Methylglutaconic Aciduria" or "cor Triatriatum" or Dextrocardi* or dextroversion or "dextro rotation" or dextrorotation or dextroposition or "Ciliary Dyskinesi*" or Levocardia or Laevocardia or "univentricular heart*" or "monoventricular heart*" or "single ventric*" or "Truncus Arterios*" or "Interrupted aortic arch*" or "ventricular inversion*") or<br>AB (CHD or CHDs or CCHD or CCHDs or "22q11.2DS" or DiGeorge* or "velofacial hypoplasia" or ALCAPA or ARVD-C or "3-Methylglutaconicaciduria" or "3-Methylglutaconic Aciduria" or "cor Triatriatum" or Dextrocardi* or dextroversion or "dextro rotation" or dextrorotation or dextroposition or "Ciliary Dyskinesi*" or Levocardia or Laevocardia or "univentricular heart*" or "monoventricular heart*" or "single ventric*" or "Truncus Arterios*" or "Interrupted aortic arch*" or "ventricular inversion*")	11337
S7	TI (cyanotic N1 (defect* or disease*)) or<br>AB (cyanotic N1 (defect* or disease*))	202
S8	TI ((ebstein* or "Taussig Bing*") N2 (sequence* or anomal* or malform* or abnorm* or disease)) or<br>AB ((ebstein* or "Taussig Bing*") N2 (sequence* or anomal* or malform* or abnorm* or disease))	463
S9	TI ((Anomal* or abnormal*) N1 (coronar* or aortic or aorta* or aorto* or venous)) or<br>AB ((Anomal* ornormal*) N1 (coronar* or aortic or aorta* or aorto* or venous))	2745

S10	TI (dysplasia N1 (Arteriohepatic or Ventricular)) or<br>AB (dysplasia N1 (Arteriohepatic or Ventricular))	478
S11	TI ((aortic or aorta or aorto) N2 (Coarctation* or fistula*)) or<br>AB ((aortic or aorta or aorto) N2 (Coarctation* or fistula*))	1529
S12	TI ((Aortoventricular or Aorticoventricular or "Aortic* Ventricular" or "Aorto* Ventricular") N1 tunnel*) or<br>AB ((Aortoventricular or Aorticoventricular or "Aortic* Ventricular" or "Aorto* Ventricular") N1 tunnel*)	9
S13	TI ((ductular or "left heart*") N1 hypoplas*) or<br>AB ((ductular or "left heart*") N1 hypoplas*)	881
S14	TI ((Bicuspid or Quadricuspid) N1 (valve* or aortic)) or<br>AB ((Bicuspid or Quadricuspid) N1 (valve* or aortic))	1292
S15	TI (atrium* N1 subdivided) or<br>AB (atrium* N1 subdivided)	1254
S16	TI (myocardial N1 (bridge* or bridging*)) or<br>AB (myocardial N1 (bridge* or bridging*))	327
S17	TI ((crisscross or "criss cross" or Triatrial) N1 heart*) or<br>AB ((crisscross or "criss cross" or Triatrial) N1 heart*)	21
S18	TI (Kartagener* N1 triad*) or<br>AB (Kartagener* N1 triad*)	1
S19	TI (Bronchiectas* N1 Polynesian) or<br>AB (Bronchiectas* N1 Polynesian)	30
S20	TI ((paten* or ligat* or occlude* or persisten* or obliteration) N2 ("ductus arterios*" or "truncus arterios*" or oval* or "ductus botalli")) or<br>AB ((paten* or ligat* or occlude* or persisten* or obliteration) N2 ("ductus arterios*" or "truncus arterios*" or oval* or "ductus botalli"))	4171
S21	TI (((Cordis or heart* or cardiac) N0 Ectop*) or ectocardia or exocardia) or<br>AB (((Cordis or heart* or cardiac) N0 Ectop*) or ectocardia or exocardia)	76
S22	TI (Eisenmenger* N0 (complex or disease or tetralogy)) or<br>AB (Eisenmenger* N0 (complex or disease or tetralogy))	11
S23	TI ((heart or cardiac or aortopulmonary or aorticopulmonary or atrial or atrium or interatrial or interauricular or ventricle or ventricular or intraventricular or interventricular or atrioventricular) N1 (septal or septum) N1 defect*) or<br>AB ((heart or cardiac or aortopulmonary or aorticopulmonary or atrial or atrium or interatrial or interauricular or ventricle or ventricular or intraventricular or interventricular or atrioventricular) N1 (septal or septum) N1 defect*)	4220
S24	TI ((endocardial or atrioventricular) N1 Cushion N1 Defect*) or<br>AB ((endocardial or atrioventricular) N1 Cushion N1 Defect*)	38
S25	TI (AVSD or "ostium primum ASD*" or "unrepaired secundum ASD*" or VSD or "persistent ostium secundum" or "patent ostium secundum") or<br>AB (AVSD or "ostium primum ASD*" or "unrepaired secundum ASD*" or VSD or "persistent ostium secundum" or "patent ostium secundum")	869

S26	TI (("double-outlet" or single) N0 (right or left) N2 ventric*) or<br>AB (("double-outlet" or single) N0 (right or left) N2 ventric*)	276
S27	TI (Heterotax* N1 visceral) or<br>AB (Heterotax* N1 visceral)	14
S28	TI (situs N1 ambigu*) or<br>AB (situs N1 ambigu*)	29
S29	TI ("atrial isomerism*" N1 (left or right)) or<br>AB ("atrial isomerism*" N1 (left or right))	65
S30	TI ((tetralogy or trilogy) N1 fallot*) or<br>AB ((tetralogy or trilogy) N1 fallot*)	1867
S31	TI ((transposition* N2 ("great arter*" or "great vessel*" or "large vessel*")) or (Dextrotransposition* or D-TGA* or Dextro-TGA* or "dexto-transposition*" or "d-transposition*" or "dextro-looped TGA*" or CCTGA* or CC-TGA* or Levotransposition* or "Levo-transposition*" or "L-looped transposition*or Levo-TGA*" or "L-TGA*")) or<br>AB ((transposition* N2 ("great arter*" or "great vessel*" or "large vessel*")) or (Dextrotransposition* or D-TGA* or Dextro-TGA* or "dexto-transposition*" or "d-transposition*" or "dextro-looped TGA*" or CCTGA* or CC-TGA* or Levotransposition* or "Levo-transposition*" or "L-looped transposition*or Levo-TGA*" or "L-TGA*"))	1178
S32	TI ((Mitral or tricuspid or pulmonary or "right atrioventricular" or "right av") N2 atresi*) or<br>AB ((Mitral or tricuspid or pulmonary or "right atrioventricular" or "right av") N2 atresi*)	670
S33	TI (congenital and "ventricular outflow obstruction*") or<br>AB (congenital and "ventricular outflow obstruction*")	13
S34	TI (("Pulmonary valve*" or "Peripheral pulmonary" or Subvalvar or Supravalvar or subvalvular or supravalvular or subaortic) N2 (regurgitation* or stenos*)) or<br>AB (("Pulmonary valve*" or "Peripheral pulmonary" or Subvalvar or Supravalvar or subvalvular or supravalvular or subaortic) N2 (regurgitation* or stenos*))	494
S35	TI (sinus N1 (Valsalva or aort* or veno*) N2 (fistula* or aneurysm* or aneurism* or defect*)) or<br>AB (sinus N1 (Valsalva or aort* or veno*) N2 (fistula* or aneurysm* or aneurism* or defect*))	488
S36	TI (straddling N1 "atrioventricular valve*") or<br>AB (straddling N1 "atrioventricular valve*")	1
S37	TI ((heart or cardiac or intercardiac or intracardiac or intercardial or interventricular or ventriculoatrial or atrioventricular or left or right) N2 shunt) or<br>AB ((heart or cardiac or intercardiac or intracardiac or intercardial or interventricular or ventriculoatrial or atrioventricular or left or right) N2 shunt)	1334
S38	TI ((Common or Persistent) N1 "atrioventricular canal") or	9

	AB ((Common or Persistent) N1 "atrioventricular canal")	
S39	TI ("Ventricular noncompaction" or "Ventricular non-compaction" or INVM or LVNC) or<br>AB ("Ventricular noncompaction" or "Ventricular non-compaction" or INVM or LVNC)	557
S40	S1 OR S2 OR S3 OR S4 OR S5 OR S6 OR S7 OR S8 OR S9 OR S10 OR S11 OR S12 OR S13 OR S14 OR S15 OR S16 OR S17 OR S18 OR S19 OR S20 OR S21 OR S22 OR S23 OR S24 OR S25 OR S26 OR S27 OR S28 OR S29 OR S30 OR S31 OR S32 OR S33 OR S34 OR S35 OR S36 OR S37 OR S38 OR S39	52626
S41	(MH "Telehealth+") or (MH "Digital Health+")	45440
S42	TI ("e-health*" or ehealth* or "m-health*" or mhealth* or telehealth* or "tele-health*" or ((mobile or remote or digital or online or virtual) N1 health*)) OR<br>AB ("e-health*" or ehealth* or "m-health*" or mhealth* or telehealth* or "tele-health*" or ((mobile or remote or digital or online or virtual) N1 health*))	25857
S43	TI (e-medic* or emedic* or telemedic* or tele-medic* or "virtual medic*" or "digital medicine" or "online medicine") or<br>AB (e-medic* or emedic* or telemedic* or tele-medic* or "virtual medic*" or "digital medicine" or "online medicine")	9178
S44	TI (telecardiology or "tele-cardiology") OR<br>AB (telecardiology or "tele-cardiology")	81
S45	TI (telecare* or "tele-care*" or "e-care*" or ehomecare* or "e-homecare*" or "e-home-care*" or ((remote or virtual or digital) W0 (care* or homecare* or "home-care*"))) OR<br>AB (telecare* or "tele-care*" or "e-care*" or ehomecare* or "e-homecare*" or "e-home-care*" or ((remote or virtual or digital) W0 (care* or homecare* or "home-care*")))	1681
S46	TI (telenursing or "tele-nursing" or ((virtual or digital or online or remote) N1 nursing)) OR<br>AB (telenursing or "tele-nursing" or ((virtual or digital or online or remote) N1 nursing))	1222
S47	TI (teleconsultation* or "tele-consultation*" or cyberconsultation* or "cyber-consultation*" or "e-consultation*" or econsultation* or videoconsultation* or ((remote or electronic or email or internet or online or web or webbased or video) N1 consultation*)) OR<br>AB (teleconsultation* or "tele-consultation*" or cyberconsultation* or "cyber-consultation*" or "e-consultation*" or econsultation* or videoconsultation* or ((remote or electronic or email or internet or online or web or webbased or video) N1 consultation*))	2061
S48	TI (telemonitor* or "tele-monitor*" or "tele-surveillance" or telesurveillance or ((distant or remote or virtual* or electronic or digital*) N1 (monitor* or surveillance))) OR<br>AB (telemonitor* or "tele-monitor*" or "tele-surveillance" or telesurveillance or ((distant or remote or virtual* or electronic or digital*) N1 (monitor* or surveillance)))	5495

S49	TI (telerehabilitation* or "tele-rehabilitation*" or "e-rehabilitation*" or ((remote or electronic or digital or virtual or email or internet or online or web or webbased or video) N1 rehabilitation*)) OR<br>AB (telerehabilitation* or "tele-rehabilitation*" or "e-rehabilitation*" or ((remote or electronic or digital or virtual or email or internet or online or web or webbased or video) N1 rehabilitation*))	1334
S50	TI (tele-psychiatry or telepsychiatry or telepsychology or tele-psychology or teletherapy or tele-therapy or ((remote or electronic or virtual or "email-based" or email or internet or "internet-based" or online or web or "web-based" or webbased or video) W0 (therapy or psychiatry or psychology))) OR<br>AB (tele-psychiatry or telepsychiatry or telepsychology or tele-psychology or teletherapy or tele-therapy or ((remote or electronic or virtual or "email-based" or email or internet or "internet-based" or online or web or "web-based" or webbased or video) W0 (therapy or psychiatry or psychology)))	1068
S51	(MH "Internet-Based Intervention")	736
S52	TI ("web-based intervention*" or "webbased intervention*" or "internet-based intervention*" or "internet intervention*" or "online-based intervention*" or "online-intervention*" or "web intervention*" or "digital intervention*" or "digital-based intervention*") OR<br>AB ("web-based intervention*" or "webbased intervention*" or "internet-based intervention*" or "internet intervention*" or "online-based intervention*" or "online-intervention*" or "web intervention*" or "digital intervention*" or "digital-based intervention*")	2668
S53	TI ((treatment N2 internet) or ((digital or "digital-based" or online or "online-based") W0 treatment)) OR<br>AB ((treatment N2 internet) or ((digital or "digital-based" or online or "online-based") W0 treatment))	586
S54	TI (virtual N1 (hospital* or ward*)) OR<br>AB (virtual N1 (hospital* or ward*))	208
S55	S41 OR S42 OR S43 OR S44 OR S45 OR S46 OR S47 OR S48 OR S49 OR S50 OR S51 OR S52 OR S53 OR S54	73423
S56	(MH "Microcomputers+")	12034
S57	(MH "Cellular Phone")	2280
S58	(MH "Wearable Sensors+") or (MH "Fitness Trackers")	7363
S59	TI ("cell-phone*" or cellphone* or smartphone* or "smart-phone*" or ((cellular or mobile) W0 (phone* or telephone*))) OR<br>AB ("cell-phone*" or cellphone* or smartphone* or "smart-phone*" or ((cellular or mobile) W0 (phone* or telephone*)))	15828
S60	TI (ipad or "mobile device*" or "palm-pilot*" or palmpilot* or "personal digital assistant*" or touchscreen* or touch-screen* or ((handheld or "hand-held" or palm* or pda or tablet or pocket) N0 computer*)) OR<br>AB (ipad or "mobile device*" or "palm-pilot*" or palmpilot* or "personal digital assistant*" or touchscreen* or touch-screen* or ((handheld or "hand-held" or palm* or pda or tablet or pocket) N0 computer*))	6211

S61	TI (smarthome* or "smart-home*") OR<br>AB (smarthome* or "smart-home*")	289
S62	TI (((wearable or wireless) N1 (technolog* or electronic* or device*)) or "fitness tracker*" or "activity tracker*" or "smart glass*" or pedometer* or smartwatch* or "smart-watch*" or healthwatch* or "health-watch*") OR<br>AB (((wearable or wireless) N1 (technolog* or electronic* or device*)) or "fitness tracker*" or "activity tracker*" or "smart glass*" or pedometer* or smartwatch* or "smart-watch*" or healthwatch* or "health-watch*")	5560
S63	S56 OR S57 OR S58 OR S59 OR S60 OR S61 OR S62	39725
S64	(MH "Wireless Communications") OR (MH "Telemetry") OR (MH "Telecommunications") OR (MH "Email") OR (MH "Instant Messaging") OR (MH "Teleconferencing") OR (MH "Telefacsimile") OR (MH "Text Messaging") OR (MH "Videoconferencing")	31284
S65	(MH "Internet") OR (MH "Social Media+")	74986
S66	(MH "Mobile Applications")	12041
S67	(MH "Virtual Reality+") OR (MH "Gamification") OR (MH "Interactive Voice Response Systems")	7979
S68	TI (wireless N0 communication*) OR<br>AB (wireless N0 communication*)	204
S69	TI (telemetr* or "remote sensing") OR<br>AB (telemetr* or "remote sensing")	2176
S70	TI (telecommunication* or tele-conferenc* or teleconferenc* or videoconferenc* or "video-conferenc*" or "text messag*" or textmessag* or texting or "short message service*" or sms or "electronic mail*" or email* or "e-mail*") OR<br>AB (telecommunication* or tele-conferenc* or teleconferenc* or videoconferenc* or "video-conferenc*" or "text messag*" or textmessag* or texting or "short message service*" or sms or "electronic mail*" or email* or "e-mail*")	23717
S71	TI "social medi*" OR<br>AB "social medi*"	17821
S72	TI gamification or<br>AB gamification	551
S73	TI ("virtual reality" or avatar*) OR<br>AB ("virtual reality" or avatar*)	6510
S74	TI (app or apps or "app-based" or (application* N1 (mobile or telephone* or web or internet or tablet))) OR<br>AB (app or apps or "app-based" or (application* N1 (mobile or telephone* or web or internet or tablet)))	17414
S75	TI (((digital or virtual or robot*) N1 (assistant*1 or companion*)) or (robot* N1 social*)) OR<br>AB (((digital or virtual or robot*) N1 (assistant*1 or companion*)) or (robot* N1 social*))	337

S76	S64 OR S65 OR S66 OR S67 OR S68 OR S69 OR S70 OR S71 OR S72 OR S73 OR S74 OR S75	157,518
S77	S55 OR S63 OR S76	233880
S78	S40 AND S77	463
S79	S40 AND S77 Limiters - Published Date: 20100101-	397
S80	S40 AND S77 Limiters - Published Date: 20100101-20231231; Publication Type: Commentary, Editorial, Letter	17
S81	S79 NOT S80	380
S82	S79 NOT S80 Limiters - Language: Danish, English, Norwegian, Spanish, Swedish	377

ERIC

S1	SU "Heart Disorders"
S2	TI (heart* N1 (abnormal* or anomal* or malform*)) or<br>AB (heart* N1 (abnormal* or anomal* or malform*))
S3	TI (congenital N1 (heart* or valve* or mitral* or aort* or tricuspid) N1 (defect* or disease* or abnormal* or anomal* or malform*)) or<br>AB (congenital N1 (heart* or valve* or mitral* or aort* or tricuspid) N1 (defect* or disease* or abnormal* or anomal* or malform*))
S4	TI (Syndrome* N1 (22q11 or "22q11.2" or "Pharyngeal Pouch" or "Autosomal Dominant Opitz G-Bbb" or "Thymic Aplasia*" or "Thymic Hypoplasia*" or Velocardiofacial or "Velo Cardio Facial" or VCF or Sedlackova* or Shprintzen* or "Conotruncal Anomaly Face" or CTAF or Alagille* or Cardiovertebral or Hepatofacioneurocardiovertebral or "Watson Miller*" or Barth* or "Bland White Garland" or Kartagener* or Siewert* or Eisenmenger* or Lutembacher* or Heterotaxy or Polysplenia* or Asplenia* or Ivemark* or "Left Heart" or LEOPARD* or "Cardio-Cutaneous" or Lentigine* or Noonan* or "Long QT" or Andersen* or "Lange Nielsen*" or Jervell* or "Cardio Auditory Syncope" or "Surdo Cardiac" or "Romano Ward*" or "Ward Romano*" or Marfan* or Turner* or Patau* or "Chromosome 13" or "Trisomy 13" or "Trisomy 18" or "Trisomy E" or Edward* or "Bonnevie Ullrich*" or "Wolf* Parkinson White*" or "Auriculoventricular Accessory Pathway" or WPW or "Anomalous Ventricular Excitation" or "False Bundle-Branch Block")) or<br>AB (Syndrome* N1 (22q11 or "22q11.2" or "Pharyngeal Pouch" or "Autosomal Dominant Opitz G-Bbb" or "Thymic Aplasia*" or "Thymic Hypoplasia*" or Velocardiofacial or "Velo Cardio Facial" or VCF or Sedlackova* or Shprintzen* or "Conotruncal Anomaly Face" or CTAF or Alagille* or Cardiovertebral or Hepatofacioneurocardiovertebral or "Watson Miller*" or Barth* or "Bland White Garland" or Kartagener* or Siewert* or Eisenmenger* or Lutembacher* or Heterotaxy or Polysplenia* or Asplenia* or Ivemark* or "Left Heart" or LEOPARD* or "Cardio-Cutaneous" or Lentigine* or Noonan* or "Long QT" or Andersen* or "Lange Nielsen*" or Jervell* or "Cardio Auditory Syncope" or "Surdo Cardiac" or "Romano Ward*" or "Ward Romano*" or Marfan* or Turner* or Patau* or "Chromosome 13" or "Trisomy 13" or "Trisomy 18" or "Trisomy E" or Edward* or "Bonnevie Ullrich*" or "Wolf* Parkinson White*" or "Auriculoventricular Accessory Pathway" or WPW or

	"Anomalous Ventricular Excitation" or "False Bundle-Branch Block"))
S5	TI (deletion N1 (22q11 or "22q11.2")) or<br>AB (deletion N1 (22q11 or "22q11.2"))
S6	TI (CHD or CHDs or CCHD or CCHDs or "22q11.2DS" or DiGeorge* or "velofacial hypoplasia" or ALCAPA or ARVD-C or "3-Methylglutaconicaciduria" or "3-Methylglutaconic Aciduria" or "cor Triatriatum" or Dextrocardi* or dextroversion or "dextro rotation" or dextrorotation or dextroposition or "Ciliary Dyskinesi*" or Levocardia or Laevocardia or "univentricular heart*" or "monoventricular heart*" or "single ventric*" or "Truncus Arterios*" or "Interrupted aortic arch*" or "ventricular inversion*") or<br>AB (CHD or CHDs or CCHD or CCHDs or "22q11.2DS" or DiGeorge* or "velofacial hypoplasia" or ALCAPA or ARVD-C or "3-Methylglutaconicaciduria" or "3-Methylglutaconic Aciduria" or "cor Triatriatum" or Dextrocardi* or dextroversion or "dextro rotation" or dextrorotation or dextroposition or "Ciliary Dyskinesi*" or Levocardia or Laevocardia or "univentricular heart*" or "monoventricular heart*" or "single ventric*" or "Truncus Arterios*" or "Interrupted aortic arch*" or "ventricular inversion*")
S7	TI (cyanotic N1 (defect* or disease*)) or<br>AB (cyanotic N1 (defect* or disease*))
S8	TI ((ebstein* or "Taussig Bing*") N2 (sequence* or anomal* or malform* or abnorm* or disease)) or<br>AB ((ebstein* or "Taussig Bing*") N2 (sequence* or anomal* or malform* or abnorm* or disease))
S9	TI ((Anomal* or abnormal*) N1 (coronar* or aortic or aorta* or aorto* or venous)) or<br>AB ((Anomal* or abnormal*) N1 (coronar* or aortic or aorta* or aorto* or venous))
S10	TI (dysplasia N1 (Arteriohepatic or Ventricular)) or<br>AB (dysplasia N1 (Arteriohepatic or Ventricular))
S11	TI ((aortic or aorta or aorto) N2 (Coarctation* or fistula*)) or<br>AB ((aortic or aorta or aorto) N2 (Coarctation* or fistula*))
S12	TI ((Aortoventricular or Aorticoventricular or "Aortic* Ventricular" or "Aorto* Ventricular") N1 tunnel*) or<br>AB ((Aortoventricular or Aorticoventricular or "Aortic* Ventricular" or "Aorto* Ventricular") N1 tunnel*)
S13	TI ((ductular or "left heart*") N1 hypoplas*) or<br>AB ((ductular or "left heart*") N1 hypoplas*)
S14	TI ((Bicuspid or Quadricuspid) N1 (valve* or aortic)) or<br>AB ((Bicuspid or Quadricuspid) N1 (valve* or aortic))
S15	TI (atrium* N1 subdivided) or<br>AB (atrium* N1 subdivided)
S16	TI (myocardial N1 (bridge* or bridging*)) or<br>AB (myocardial N1 (bridge* or bridging*))
S17	TI ((crisscross or "criss cross" or Triatrial) N1 heart*) or<br>AB ((crisscross or "criss cross" or Triatrial) N1 heart*)

S18	TI (Kartagener* N1 triad*) or<br>AB (Kartagener* N1 triad*)
S19	TI (Bronchiectas* N1 Polynesian) or<br>AB (Bronchiectas* N1 Polynesian)
S20	TI ((paten* or ligat* or occlude* or persisten* or obliteration) N2 ("ductus arterios*" or "truncus arterios*" or oval* or "ductus botalli")) or<br>AB ((paten* or ligat* or occlude* or persisten* or obliteration) N2 ("ductus arterios*" or "truncus arterios*" or oval* or "ductus botalli"))
S21	TI (((Cordis or heart* or cardiac) N0 Ectop*) or ectocardia or exocardia) or<br>AB (((Cordis or heart* or cardiac) N0 Ectop*) or ectocardia or exocardia)
S22	TI (Eisenmenger* N0 (complex or disease or tetralogy)) or<br>AB (Eisenmenger* N0 (complex or disease or tetralogy))
S23	TI ((heart or cardiac or aortopulmonary or aorticopulmonary or atrial or atrium or interatrial or interauricular or ventricle or ventricular or intraventricular or interventricular or atrioventricular) N1 (septal or septum) N1 defect*) or<br>AB ((heart or cardiac or aortopulmonary or aorticopulmonary or atrial or atrium or interatrial or interauricular or ventricle or ventricular or intraventricular or interventricular or atrioventricular) N1 (septal or septum) N1 defect*)
S24	TI ((endocardial or atrioventricular) N1 Cushion N1 Defect*) or<br>AB ((endocardial or atrioventricular) N1 Cushion N1 Defect*)
S25	TI (AVSD or "ostium primum ASD*" or "unrepaired secundum ASD*" or VSD or "persistent ostium secundum" or "patent ostium secundum") or<br>AB (AVSD or "ostium primum ASD*" or "unrepaired secundum ASD*" or VSD or "persistent ostium secundum" or "patent ostium secundum")
S26	TI (("double-outlet" or single) N0 (right or left) N2 ventric*) or<br>AB (("double-outlet" or single) N0 (right or left) N2 ventric*)
S27	TI (Heterotax* N1 visceral) or<br>AB (Heterotax* N1 visceral)
S28	TI (situs N1 ambigu*) or<br>AB (situs N1 ambigu*)
S29	TI ("atrial isomerism*" N1 (left or right)) or<br>AB ("atrial isomerism*" N1 (left or right))
S30	TI ((tetralogy or trilogy) N1 fallot*) or<br>AB ((tetralogy or trilogy) N1 fallot*)
S31	TI ((transposition* N2 ("great arter*" or "great vessel*" or "large vessel*")) or (Dextrotransposition* or D-TGA* or Dextro-TGA* or "dexto-transposition*" or "d-transposition*" or "dextro-looped TGA*" or CCTGA* or CC-TGA* or Levotransposition* or "Levo-transposition*" or "L-looped transposition*or Levo-TGA*" or "L-TGA*")) or<br>AB ((transposition* N2 ("great arter*" or "great vessel*" or "large vessel*")) or (Dextrotransposition* or D-TGA* or Dextro-TGA* or "dexto-transposition*" or "d-transposition*" or "dextro-looped TGA*" or CCTGA* or CC-TGA* or Levotransposition* or "Levo-transposition*" or "L-looped transposition*or Levo-TGA*" or "L-TGA*"))

S32	TI ((Mitral or tricuspid or pulmonary or "right atrioventricular" or "right av") N2 atresi*) or<br>AB ((Mitral or tricuspid or pulmonary or "right atrioventricular" or "right av") N2 atresi*)
S33	TI (congenital and "ventricular outflow obstruction*") or<br>AB (congenital and "ventricular outflow obstruction*")
S34	TI (("Pulmonary valve*" or "Peripheral pulmonary" or Subvalvar or Supravalvar or subvalvular or supravalvular or subaortic) N2 (regurgitation* or stenos*)) or<br>AB (("Pulmonary valve*" or "Peripheral pulmonary" or Subvalvar or Supravalvar or subvalvular or supravalvular or subaortic) N2 (regurgitation* or stenos*))
S35	TI (sinus N1 (Valsalva or aort* or veno*) N2 (fistula* or aneurysm* or aneurism* or defect*)) or<br>AB (sinus N1 (Valsalva or aort* or veno*) N2 (fistula* or aneurysm* or aneurism* or defect*))
S36	TI (straddling N1 "atrioventricular valve*") or<br>AB (straddling N1 "atrioventricular valve*")
S37	TI ((heart or cardiac or intercardiac or intracardiac or intercardial or interventricular or ventriculoatrial or atrioventricular or left or right) N2 shunt) or<br>AB ((heart or cardiac or intercardiac or intracardiac or intercardial or interventricular or ventriculoatrial or atrioventricular or left or right) N2 shunt)
S38	TI ((Common or Persistent) N1 "atrioventricular canal") or<br>AB ((Common or Persistent) N1 "atrioventricular canal")
S39	TI ("Ventricular noncompaction" or "Ventricular non-compaction" or INVM or LVNC) or<br>AB ("Ventricular noncompaction" or "Ventricular non-compaction" or INVM or LVNC)
S40	S1 OR S2 OR S3 OR S4 OR S5 OR S6 OR S7 OR S8 OR S9 OR S10 OR S11 OR S12 OR S13 OR S14 OR S15 OR S16 OR S17 OR S18 OR S19 OR S20 OR S21 OR S22 OR S23 OR S24 OR S25 OR S26 OR S27 OR S28 OR S29 OR S30 OR S31 OR S32 OR S33 OR S34 OR S35 OR S36 OR S37 OR S38 OR S39
S41	TI ("e-health*" or ehealth* or "m-health*" or mhealth* or telehealth* or "tele-health*" or ((mobile or remote or digital or online or virtual) N1 health*)) OR<br>AB ("e-health*" or ehealth* or "m-health*" or mhealth* or telehealth* or "tele-health*" or ((mobile or remote or digital or online or virtual) N1 health*))
S42	TI (e-medic* or emedic* or telemedic* or tele-medic* or "virtual medic*" or "digital medicine" or "online medicine") or<br>AB (e-medic* or emedic* or telemedic* or tele-medic* or "virtual medic*" or "digital medicine" or "online medicine")
S43	TI (telecardiology or "tele-cardiology") OR<br>AB (telecardiology or "tele-cardiology")
S44	TI (telecare* or "tele-care*" or "e-care*" or ehomecare* or "e-homecare*" or "e-home-care*" or ((remote or virtual or digital) W0 (care* or homecare* or "home-care*"))) OR<br>AB (telecare* or "tele-care*" or "e-care*" or ehomecare* or "e-homecare*" or "e-home-care*" or ((remote or virtual or digital) W0 (care* or homecare* or "home-care*")))
S45	TI (telenursing or "tele-nursing" or ((virtual or digital or online or remote) N1 nursing))

	OR<br>AB (telenursing or "tele-nursing" or ((virtual or digital or online or remote) N1 nursing))
S46	TI (teleconsultation* or "tele-consultation*" or cyberconsultation* or "cyber-consultation*" or "e-consultation*" or econsultation* or videoconsultation* or ((remote or electronic or email or internet or online or web or webbased or video) N1 consultation*)) OR<br>AB (teleconsultation* or "tele-consultation*" or cyberconsultation* or "cyber-consultation*" or "e-consultation*" or econsultation* or videoconsultation* or ((remote or electronic or email or internet or online or web or webbased or video) N1 consultation*))
S47	TI (telemonitor* or "tele-monitor*" or "tele-surveillance" or telesurveillance or ((distant or remote or virtual* or electronic or digital*) N1 (monitor* or surveillance))) OR<br>AB (telemonitor* or "tele-monitor*" or "tele-surveillance" or telesurveillance or ((distant or remote or virtual* or electronic or digital*) N1 (monitor* or surveillance)))
S48	TI (telerehabilitation* or "tele-rehabilitation*" or "e-rehabilitation*" or ((remote or electronic or digital or virtual or email or internet or online or web or webbased or video) N1 rehabilitation*)) OR<br>AB (telerehabilitation* or "tele-rehabilitation*" or "e-rehabilitation*" or ((remote or electronic or digital or virtual or email or internet or online or web or webbased or video) N1 rehabilitation*))
S49	TI (tele-psychiatry or telepsychiatry or telepsychology or tele-psychology or teletherapy or tele-therapy or ((remote or electronic or virtual or "email-based" or email or internet or "internet-based" or online or web or "web-based" or webbased or video) W0 (therapy or psychiatry or psychology))) OR<br>AB (tele-psychiatry or telepsychiatry or telepsychology or tele-psychology or teletherapy or tele-therapy or ((remote or electronic or virtual or "email-based" or email or internet or "internet-based" or online or web or "web-based" or webbased or video) W0 (therapy or psychiatry or psychology)))
S50	TI ("web-based intervention*" or "webbased intervention*" or "internet-based intervention*" or "internet intervention*" or "online-based intervention*" or "online-intervention*" or "web intervention*" or "digital intervention*" or "digital-based intervention*") OR<br>AB ("web-based intervention*" or "webbased intervention*" or "internet-based intervention*" or "internet intervention*" or "online-based intervention*" or "online-intervention*" or "web intervention*" or "digital intervention*" or "digital-based intervention*")
S51	TI ((treatment N2 internet) or ((digital or "digital-based" or online or "online-based") W0 treatment)) OR<br>AB ((treatment N2 internet) or ((digital or "digital-based" or online or "online-based") W0 treatment))
S52	TI (virtual N1 (hospital* or ward*)) OR<br>AB (virtual N1 (hospital* or ward*))
S53	S41 OR S42 OR S43 OR S44 OR S45 OR S46 OR S47 OR S48 OR S49 OR S50 OR S51 OR S52
S54	DE ("Computers" OR "Laptop Computers" OR "Handheld Devices")

S55	TI ("cell-phone*" or cellphone* or smartphone* or "smart-phone*" or ((cellular or mobile) W0 (phone* or telephone*))) OR<br>AB ("cell-phone*" or cellphone* or smartphone* or "smart-phone*" or ((cellular or mobile) W0 (phone* or telephone*)))
S56	TI (ipad or "mobile device*" or "palm-pilot*" or palmpilot* or "personal digital assistant*" or touchscreen* or touch-screen* or ((handheld or "hand-held" or palm* or pda or tablet or pocket) N0 computer*)) OR<br>AB (ipad or "mobile device*" or "palm-pilot*" or palmpilot* or "personal digital assistant*" or touchscreen* or touch-screen* or ((handheld or "hand-held" or palm* or pda or tablet or pocket) N0 computer*))
S57	TI (smarthome* or "smart-home*") OR<br>AB (smarthome* or "smart-home*")
S58	TI (((wearable or wireless) N1 (technolog* or electronic* or device*)) or "fitness tracker*" or "activity tracker*" or "smart glass*" or pedometer* or smartwatch* or "smart-watch*" or healthwatch* or "health-watch*") OR<br>AB (((wearable or wireless) N1 (technolog* or electronic* or device*)) or "fitness tracker*" or "activity tracker*" or "smart glass*" or pedometer* or smartwatch* or "smart-watch*" or healthwatch* or "health-watch*")
S59	S54 OR S55 OR S56 OR S57 OR S58
S60	DE ("Teleconferencing" OR "Videoconferencing" OR "Synchronous Communication" OR "Electronic Mail" OR "Telecommunications" or "Computer Assisted Instruction" OR "Computer Mediated Communication")
S61	DE ("Internet" OR "Social Media")
S62	TI (wireless N0 communication*) OR<br>AB (wireless N0 communication*)
S63	TI (telemetr* or "remote sensing") OR<br>AB (telemetr* or "remote sensing")
S64	TI (telecommunication* or tele-conferenc* or teleconferenc* or videoconferenc* or "video-conferenc*" or "text messag*" or textmessag* or texting or "short message service*" or sms or "electronic mail*" or email* or "e-mail*") OR<br>AB (telecommunication* or tele-conferenc* or teleconferenc* or videoconferenc* or "video-conferenc*" or "text messag*" or textmessag* or texting or "short message service*" or sms or "electronic mail*" or email* or "e-mail*")
S65	TI "social medi*" OR<br>AB"social medi*"
S66	TI gamification or<br>ABgamification
S67	TI ("virtual reality" or avatar*) OR<br>AB ("virtual reality" or avatar*)
S68	TI (app or apps or "app-based" or (application* N1 (mobile or telephone* or web or internet or tablet))) OR<br>AB (app or apps or "app-based" or (application* N1 (mobile or telephone* or web or internet or tablet)))

S69	TI (((digital or virtual or robot*) N1 (assistant*1 or companion*)) or (robot* N1 social*)) OR<br>AB (((digital or virtual or robot*) N1 (assistant*1 or companion*)) or (robot* N1 social*))
S70	S60 OR S61 OR S62 OR S63 OR S64 OR S65 OR S66 OR S67 OR S68 OR S69
S71	S53 OR S59 OR S70
S72	S40 AND S71
S73	S40 AND S71<br>Limiters - Date Published: 20100101-

Web of Science Core collection

1	TS= ("Ventricular noncompaction" or "Ventricular non-compaction" or INVM or LVNC)
2	TS= ((Common or Persistent) NEAR/1 "atrioventricular canal")
3	TS= ((heart or cardiac or intercardiac or intracardiac or intercardial or interventricular or ventriculoatrial or atrioventricular or left or right) NEAR/2 shunt)
4	TS= (straddling NEAR/1 "atrioventricular valve*")
5	TS= (sinus NEAR/1 (Valsalva or aort* or veno*) NEAR/2 (fistula* or aneurysm* or aneurism* or defect*))
6	TS= (("Pulmonary valve*" or "Peripheral pulmonary" or Subvalvar or Supravalvar or subvalvular or supravalvular or subaortic) NEAR/2 (regurgitation* or stenos*))
7	TS= (congenital and "ventricular outflow obstruction*")
8	TS= ((Mitral or tricuspid or pulmonary or "right atrioventricular" or "right av") NEAR/2 atresi*)
9	TS= ((transposition* NEAR/2 ("great arter*" or "great vessel*" or "large vessel*")) or (Dextrotransposition* or D-TGA* or Dextro-TGA* or "dexto-transposition*" or "d-transposition*" or "dextro-looped TGA*" or CCTGA* or CC-TGA* or Levotransposition* or "Levo-transposition*" or "L-looped transposition*or Levo-TGA*" or "L-TGA*"))
10	TS= ((tetralogy or trilogy) NEAR/1 fallot*)
11	TS= ("atrial isomerism*" NEAR/1 (left or right))
12	TS= (situs NEAR/1 ambigu*)
13	TS= (Heterotax* NEAR/1 visceral)
14	TS= (("double-outlet" or single) NEAR/0 (right or left) NEAR/2 ventric*)
15	TS= (AVSD or "ostium primum ASD*" or "unrepaired secundum ASD*" or VSD or "persistent ostium secundum" or "patent ostium secundum")
16	TS= ((endocardial or atrioventricular) NEAR/1 Cushion NEAR/1 Defect*)
17	TS= ((heart or cardiac or aortopulmonary or aorticopulmonary or atrial or atrium or interatrial or interauricular or ventricle or ventricular or intraventricular or interventricular or atrioventricular) NEAR/1 (septal or septum) NEAR/1 defect*)

18	TS= (Eisenmenger* NEAR/0 (complex or disease or tetralogy))
19	TS= (((Cordis or heart* or cardiac) NEAR/0 Ectop*) or ectocardia or exocardia)
20	TS= ((paten* or ligat* or occlude* or persisten* or obliteration) NEAR/2 ("ductus arterios*" or "truncus arterios*" or oval* or "ductus botalli"))
21	TS= (Bronchiectas* NEAR/1 Polynesian)
22	TS= (Kartagener* NEAR/1 triad*)
23	TS= ((crisscross or "criss cross" or Triatrial) NEAR/1 heart*)
24	TS= (myocardial NEAR/1 (bridge* or bridging*))
25	TS= (atrium* NEAR/1 subdivided)
26	TS= ((Bicuspid or Quadricuspid) NEAR/1 (valve* or aortic))
27	TS= ((ductular or "left heart*") NEAR/1 hypoplas*)
28	TS= ((Aortoventricular or Aorticoventricular or "Aortic* Ventricular" or "Aorto* Ventricular") NEAR/1 tunnel*)
29	TS= ((aortic or aorta or aorto) NEAR/2 (Coarctation* or fistula*))
30	TS= (dysplasia NEAR/1 (Arteriohepatic or Ventricular))
31	TS= ((Anomal* or abnormal*) NEAR/1 (coronar* or aortic or aorta* or aorto* or venous))
32	TS= ((ebstein* or "Taussig Bing*") NEAR/2 (sequence* or anomal* or malform* or abnorm* or disease))
33	TS= (cyanotic NEAR/1 (defect* or disease*))
34	TS= (CHD or CHDs or CCHD or CCHDs or "22q11.2DS" or DiGeorge* or "velofacial hypoplasia" or ALCAPA or ARVD-C or "3-Methylglutaconicaciduria" or "3-Methylglutaconic Aciduria" or "cor Triatriatum" or Dextrocardi* or dextroversion or "dextro rotation" or dextrorotation or dextroposition or "Ciliary Dyskinesi*" or Levocardia or Laevocardia or "univentricular heart*" or "monoventricular heart*" or "single ventric*" or "Truncus Arterios*" or "Interrupted aortic arch*" or "ventricular inversion*")
35	TS= (deletion NEAR/1 (22q11 or "22q11.2"))
36	TS= (Syndrome* NEAR/1 (22q11 or "22q11.2" or "Pharyngeal Pouch" or "Autosomal Dominant Opitz G-Bbb" or "Thymic Aplasia*" or "Thymic Hypoplasia*" or Velocardiofacial or "Velo Cardio Facial" or VCF or Sedlackova* or Shprintzen* or "Conotruncal Anomaly Face" or CTAF or Alagille* or Cardiovertebral or Hepatofacioneurocardiovertebral or "Watson Miller*" or Barth* or "Bland White Garland" or Kartagener* or Siewert* or Eisenmenger* or Lutembacher* or Heterotaxy or Polysplenia* or Asplenia* or Ivemark* or "Left Heart" or LEOPARD* or "Cardio-Cutaneous" or Lentigine* or Noonan* or "Long QT" or Andersen* or "Lange Nielsen*" or Jervell* or "Cardio Auditory Syncope" or "Surdo Cardiac" or "Romano Ward*" or "Ward Romano*" or Marfan* or Turner* or Patau* or "Chromosome 13" or "Trisomy 13" or "Trisomy 18" or "Trisomy E" or Edward* or "Bonnevie Ullrich*" or "Wolf* Parkinson White*" or "Auriculoventricular Accessory Pathway" or WPW or "Anomalous Ventricular Excitation" or "False Bundle-Branch Block"))

37	TS= (congenital NEAR/1 (heart* or valve* or mitral* or aort* or tricuspid) NEAR/1 (defect* or disease* or abnormal* or anomal* or malform*))
38	TS= (heart* NEAR/1 (abnormal* or anomal* or malform*))
39	#1 OR #2 OR #3 OR #4 OR #5 OR #6 OR #7 OR #8 OR #9 OR #10 OR #11 OR #12 OR #13 OR #14 OR #15 OR #16 OR #17 OR #18 OR #19 OR #20 OR #21 OR #22 OR #23 OR #24 OR #25 OR #26 OR #27 OR #28 OR #29 OR #30 OR #31 OR #32 OR #33 OR #34 OR #35 OR #36 OR #37 OR #38
40	TS=("e-health*" or ehealth* or "m-health*" or mhealth* or telehealth* or "tele-health*" or ((mobile or remote or digital or online or virtual) NEAR/1 health*))
41	TS=(e-medic* or emedic* or telemedic* or tele-medic* or "virtual medic*" or "digital medicine" or "online medicine")
42	TS=(telecardiology or "tele-cardiology")
43	TS=(telecare* or "tele-care*" or "e-care*" or ehomecare* or "e-homecare*" or "e-home-care*" or ((remote or virtual or digital) NEAR/0 (care* or homecare* or "home-care*")))
44	TS=(telenursing or "tele-nursing" or ((virtual or digital or online or remote) NEAR/1 nursing))
45	TS=(teleconsultation* or "tele-consultation*" or cyberconsultation* or "cyber-consultation*" or "e-consultation*" or econsultation* or videoconsultation* or ((remote or electronic or email or internet or online or web or webbased or video) NEAR/1 consultation*))
46	TS=(telemonitor* or "tele-monitor*" or "tele-surveillance" or telesurveillance or ((distant or remote or virtual* or electronic or digital*) NEAR/1 (monitor* or surveillance)))
47	TS=(telerehabilitation* or "tele-rehabilitation*" or "e-rehabilitation*" or ((remote or electronic or digital or virtual or email or internet or online or web or webbased or video) NEAR/1 rehabilitation*))
48	TS=(tele-psychiatry or telepsychiatry or telepsychology or tele-psychology or teletherapy or tele-therapy or ((remote or electronic or virtual or "email-based" or email or internet or "internet-based" or online or web or "web-based" or webbased or video) NEAR/0 (therapy or psychiatry or psychology)))
49	TS=("web-based intervention*" or "webbased intervention*" or "internet-based intervention*" or "internet intervention*" or "online-based intervention*" or "online-intervention*" or "web intervention*" or "digital intervention*" or "digital-based intervention*")
50	TS=((treatment NEAR/2 internet) or ((digital or "digital-based" or online or "online-based") NEAR/0 treatment))
51	TS=(virtual NEAR/1 (hospital* or ward*))
52	#40 OR #41 OR #42 OR #43 OR #44 OR #45 OR #46 OR #47 OR #48 OR #49 OR #50 OR #51
53	TS=("cell-phone*" or cellphone* or smartphone* or "smart-phone*" or ((cellular or mobile) NEAR/0 (phone* or telephone*)))
54	TS=(ipad or "mobile device*" or "palm-pilot*" or palmpilot* or "personal digital assistant*" or touchscreen* or touch-screen* or ((handheld or "hand-held" or palm* or

	pda or tablet or pocket) NEAR/0 computer*))
55	TS=(smarthome* or "smart-home*")
56	TS=(((wearable or wireless) NEAR/1 (technolog* or electronic* or device*)) or "fitness tracker*" or "activity tracker*" or "smart glass*" or pedometer* or smartwatch* or "smart-watch*" or healthwatch* or "health-watch*")
57	#56 OR #55 OR #54 OR #53
58	TS=(wireless NEAR/0 communication*)
59	TS=(telemetr* or "remote sensing")
60	TS=(telecommunication* or tele-conferenc* or teleconferenc* or videoconferenc* or "video-conferenc*" or "text messag*" or textmessag* or texting or "short message service*" or sms or "electronic mail*" or email* or "e-mail*")
61	TS="social medi*"
62	TS=gamification
63	TS=("virtual reality" or avatar*)
64	TS=(app or apps or "app-based" or (application* NEAR/1 (mobile or telephone* or web or internet or tablet)))
65	TS=(((digital or virtual or robot*) NEAR/1 (assistant*1 or companion*)) or (robot* NEAR/1 social*))
66	#65 OR #64 OR #63 OR #62 OR #61 OR #60 OR #59 OR #58
67	#66 OR #57 OR #52
68	#67 AND #39
69	#67 AND #39 Timespan: 2010-01-01 -
70	LA=(Norwegian OR Danish OR Swedish OR Spanish OR English)
71	#70 AND #69
72	Refined By:NOT Document Types: Proceeding Paper or Editorial Material or Letter or Meeting Abstract.
