# Supplementary material for: Effectiveness of eHealth Interventions for Adolescents and Young Adults With Congenital Heart Disease: Systematic Review
Source: J Med Internet Res. 2026 Jul 8;28:e91424. doi: 10.2196/91424 (PMC13392527; doi:10.2196/91424)
Supplement: Multimedia Appendix 6 [file jmir_v28i1e91424_app6.doc]

| **Table 2.** Characteristics of eHealth interventions studies for adolescents and young adults with CHD | | | | | | | |
| --- | --- | --- | --- | --- | --- | --- | --- |
| N | Author, Year, Country of origin | Study design | Sample Size | Age Partici-pants | Intervention (content and delivery) | Theoretical perspective/  Measure | Outcomes/effectiveness of the eHealth intervention |
| 1 | Freeden-  berg et al.,  2017, USA | RCT (two-group design) | 46 | 12–18 years  (mean 14.8) | MBSR program including yoga, meditation, cognitive restructuring, and group support, delivered through structured sessions compared with a video-based online support group; group-based interventions with partial delivery via videoconferencing | HADS (anxiety, depression); RSQ (illness-related stress and coping) | Both interventions reduced illness-related stress; no significant differences between groups; MBSR participants  reported learning coping strategies (qualitative findings). |
| 2 | Jackson et al., 2022, USA | RCT (two-group design) | 60 | 15–18 years  (mean 16.3) | CHD-PAL lifestyle intervention including  videoconferencing sessions with a trained  health coach, individualized exercise prescription, and Fitbit-supported  self-monitoring; delivered remotely with ongoing interaction over approximately 20 weeks | Theory of Planned Behavior; MVPA  (accelerometer-measured) | No significant effects on MVPA in the total sample; increased MVPA among participants with low baseline activity <21 min/day). Intervention was feasible and acceptable. |
| 3 | Klausen et al., 2016, Denmark | RCT (parallel-group) | 158 | 13–16 years  (mean 14.6) | Individually tailored eHealth physical activity encouragement delivered via internet, mobile application, and SMS, including  feedback, self-monitoring, and goal-setting, alongside group-based education and individual counseling; duration 52 weeks, with no direct interaction with HCPs within the eHealth component | Social Cognitive Theory (applied in education/  counseling); VO_2_ peak  (primary outcome) | No significant effects on VO_2_ peak, physical activity, or quality of life. The eHealth intervention showed no added benefit beyond education and counseling. |
| 4 | Lin et al., 2021, Taiwan | RCT  (3-arm parallel) | 143 | 15–24 years  (mean  ~19.5) | Self-regulation theory-based mHealth intervention (COOL program) including the COOL Passport app, Health Promotion Cloud platform; features included self-monitoring, tailored feedback, gamification,  and peer interaction via an interactive platform; delivered via smartphone over 12 months, with limited interaction with HCPs | Self-regulation theory; Leuven Knowledge Questionnaire for CHD; International Physical Activity Questionnaire (IPAQ) | No significant improvement in disease knowledge or physical activity compared with control at 6 or 12 months. The intervention showed limited effectiveness despite long-term delivery. |
| 5 | Liddle et al., 2022, USA | Pre-post intervention (pros-pective) | 22 | 13-18 years  (mean  ~ 16) | Patient-specific digital 3D heart models combined with tele-education sessions delivered via videoconferencing by a pediatric cardiologist;  interactive visualization of cardiac anatomy  with real-time explanation; single session  (~30 minutes) intervention with direct interaction. | Medical knowledge assessed pre- and post-  intervention using a structured scoring system based on questionnaire responses scored by blinded cardiologists | Significant improvement in medical knowledge of cardiac defects and surgeries post-intervention (p < 0.01); increased understanding may support transition readiness. |
| 6 | Han et al., 2023, Canada | RCT (1:1 allocation) | 68 | 16–18 years | “Just TRAC it!” smartphone-based intervention using built-in features  (Notes, Calendar, Camera, Contacts) to  support self-management; combined with a one-time nurse-led education session; no ongoing digital interaction with HCPs | Self-efficacy framework; TRANSITION-Q Questionnaire | Transition readiness (TRANSITION-Q) improved over time in both groups, with no significant between-group differences; high acceptability and perceived usefulness, though engagement and privacy concerns were noted. |
| 7 | Hwang et al., 2025, South Korea | RCT (parallel-group) | 28 | 12–19 years | Multi-component online health management program including weekly Zoom-based group sessions (4x60 minutes), 1:1 telephone coaching (4 sessions), Facebook-based information sharing, and individualized dietary feedback; delivered by healthcare professionals with continuous interaction over 4 weeks; included self-monitoring of physical activity and health behaviors | Self-efficacy theory; K-SRAHP (self-efficacy); PCQLI (quality of life); ActiGraph  (physical activity and sleep) | Significant improvements in health self-efficacy, psychosocial quality of life, daily step counts, and MVPA, with reduced sedentary behavior; no significant effect on sleep outcomes. |
| 8 | Cousino et al., 2025,  USA | Pilot pre-post study (single group) | 20 | 13–18 years  (mean 16.1) | Group-based telemedicine psychoeducational intervention (WE BEAT) delivered by videoconferencing, consisting of five weekly 45-minute sessions led by a licensed psychologist; focused on resilience, coping skills, and psychological well-being | Resilience theory; Connor–Davidson Resilience Scale, Benefit and Burden Scale for Children, and NIH PROMIS measures (depressive symptoms, anxiety, peer relationships, life satisfaction, meaning, and purpose/positive affect) | The intervention was feasible and acceptable, with high attendance and satisfaction. Post-intervention, resilience increased, depressive symptoms decreased, and meaning/purpose in life improved. No meaningful changes were observed for anxiety, life satisfaction, positive affect, peer relationships, or benefit/burden of illness. |
| Abbreviations. MBSR: Mindfulness-Based Stress Reduction; HADS: Hospital Anxiety and Depression Scale; RSQ: Responses to Stress Questionnaire; CHD-PAL: Congenital Heart Disease Physical Activity Lifestyle; MVPA: moderate-to-vigorous physical activity; VO_2_; peak oxygen uptake; COOL: Care & Organize Our Lifestyle; CHD: congenital heart disease; IPAQ: International Physical Activity Questionnaire; K-SRAHP: Korean Self-Rated Abilities for Health Practices (Health Self-Efficacy Measure); PCQLI: Pediatric Cardiac Quality of Life Inventory; ActiGraph: accelerometer-based wearable device for measuring physical activity and sleep; PROMIS: Patient-Reported Outcomes Measurement Information System | | | | | | | |
