# Supplementary material for: Effectiveness of eHealth Interventions for Adolescents and Young Adults With Congenital Heart Disease: Systematic Review
Source: J Med Internet Res. 2026 Jul 8;28:e91424. doi: 10.2196/91424 (PMC13392527; doi:10.2196/91424)
Supplement: Multimedia Appendix 7 [file jmir_v28i1e91424_app7.doc]

| **Table 3.** Measured outcomes, instruments, and patient‑reported outcome measures (PROMs) in included studies | | | |
| --- | --- | --- | --- |
| Author(s) | Outcomes measured | Measurement tools/Methods | PROMS |
| Freedenberg et al. | Anxiety, depression, illness-related stress, coping | Hospital Anxiety and Depression Scale (HADS); Responses to Stress Questionnaire (RSQ) | HADS; RSQ |
| Jackson et al. | Moderate-to-vigorous physical activity (MVPA), sedentary behavior, cardiorespiratory fitness | ActiGraph accelerometer (MVPA, sedentary behavior), exercise stress test (VO_2_ peak) | None |
| Klausen et al. | Cardiorespiratory fitness (VO_2_ peak), physical activity, HRQoL | Cycle ergometer exercise test (VO_2_ peak); ActiGraph accelerometer and validated questionnaire (physical activity); PedsQL generic and disease-specific modules (HRQoL) | PedsQL (generic and disease-spec~~i~~fic modules) |
| Lin et al. | Disease knowledge, physical activity | Leuven Knowledge Questionnaire for Congenital Heart Disease (LKQCHD); International Physical Activity Questionnaire (IPAQ, Taiwan version) | Leuven Knowledge Questionnaire (LKQCHD); International Physical Activity Questionnaire (IPAQ) |
| Liddle et al. | Medical knowledge of cardiac defects and surgeries | Pre- and post-intervention questionnaires (free-text responses) scored by blinded cardiologists using a structured medical knowledge classification system | None (observer-rated outcome based on patient responses) |
| Han et al. | Transition readiness, frequency of use, and perceived usefulness of  the intervention | TRANSITION-Q Questionnaire (validated measure of self-management skills); study-specific questionnaire~~,~~ assessing frequency of use and perceived usefulness of smartphone applications | TRANSITION-Q Questionnaire |
| Hwang et al. | Health self-efficacy, health behaviors (physical activity, sedentary behavior, sleep), HRQoL | K-SRAHP (health self-efficacy), PCQLI (HRQoL), ActiGraph accelerometer  (physical activity and sleep), and self-reported sedentary behavior questionnaire | K-SRAHP and PCQLI questionnaires |
| Cousino et al. | Resilience, benefit/burden of illness, depressive symptoms, anxiety, peer relationships, and life satisfaction | Connor–Davidson Resilience Scale, Benefit/Burden Scale for Children, and NIH PROMIS questionnaires (depressive symptoms, anxiety, peer relationships,  life satisfaction) | Connor–Davidson Resilience Scale, Benefit/Burden Scale for Children, and NIH PROMIS measures |
| Abbreviations. PROMS: Patient-Reported Outcome Measures; HADS: Hospital Anxiety and Depression Scale; RSQ: Responses to Stress Questionnaire; MVPA: moderate-to-vigorous physical activity; VO_2_: peak oxygen uptake; PedsQL: Pediatric Quality of Life Inventory; K-SRAHP: Korean Self-Rated Abilities for Health Practices (Health Self-Efficacy Measure); PCQLI: Pediatric Cardiac Quality of Life Inventory; PROMIS: Patient-Reported Outcomes Measurement Information System | | | |
